# Fluoroless Workflow for Pulsed Field Ablation of Atrial Fibrillation Using the Sphere-9 and VARIPULSE Catheters

**DOI:** 10.3390/jcm15176838

**Published:** 2026-09-03

**Authors:** Rachita Kour, Anthony Costa, Shakeel Jamal, Fayaz Hakim, Hassaan Imtiaz, Khalil Kanjwal

**Affiliations:** 1 Division of Internal Medicine, Mercy Catholic Medical Center, Darby, PA 19023, USA; rachita.kaur007@gmail.com; 2 Department of Internal Medicine, Henry Ford Genesys Hospital, Grand Blanc, MI 48439, USA; acostai1@hfhs.org; 3 Division of Cardiology, Memorial Medical Center, Las Cruces, NM 88011, USA; shakeel_jamal@yahoo.com; 4 Electrophysiology Section, Henry Ford Genesys Hospital, Grand Blanc, MI 48439, USA; fhakim1@hfhs.org; 5 Department of Hospital Medicine, McLaren Bay Region, Bay City, MI 48708, USA; hassaan.imtiaz214@gmail.com

**Keywords:** atrial fibrillation, pulsed field ablation, zero fluoroscopy, Sphere-9, VARIPULSE, pulmonary vein isolation, intracardiac echocardiography, radiation exposure, steerable sheath

## Abstract

**Background:** Catheter ablation is the most effective rhythm-control strategy for symptomatic atrial fibrillation (AF). A fluoroless approach eliminates ionizing radiation exposure for patients, operators, and laboratory staff and removes the ergonomic burden of leaded protective garments. Pulsed field ablation (PFA) is a predominantly non-thermal energy modality with a distinct safety profile, now addressed in dedicated international consensus documents. Several PFA catheters are natively integrated with three-dimensional electroanatomical mapping (3D EAM) platforms and are therefore well suited to fluoroscopy-free workflows. This review is confined to two of them—Sphere-9 (Affera/Medtronic) and VARIPULSE (Biosense Webster)—with which the authors have direct procedural experience. **Objective:** This study seeks to describe a practical, expert- and experience-based approach to zero-fluoroscopy AF ablation with these two catheters, to review the harms of radiation exposure, and to synthesize the currently available and still limited evidence for fluoroless PFA. The workflow presented is explicitly not an evidence-validated or broadly generalizable algorithm, and each procedural step is labeled according to whether it rests on comparative data, device-specific studies or instructions for use, or institutional practice and expert opinion. **Methods:** This is a structured narrative review. PubMed/MEDLINE was searched to 30 June 2026 (final search 30 June 2026), with no lower date restriction, using controlled vocabulary and free-text terms for atrial fibrillation, catheter ablation, zero or minimal fluoroscopy, pulsed field ablation, Sphere-9, Affera, and VARIPULSE. English-language randomized trials, cohorts, registries, systematic reviews, and society documents reporting procedural or clinical outcomes were eligible; reference lists were hand-searched. Conference abstracts, preprints, and non-peer-reviewed manufacturer reports were excluded from all numerical statements. **Results:** Four meta-analyses report comparable acute success, arrhythmia recurrence, and complication rates between zero-fluoroscopy and fluoroscopy-guided AF ablation, with procedural feasibility of 95.1% and a crossover rate of 1.26%. These are pooled observational comparisons without pre-specified non-inferiority designs or margins, and they should not be read as establishing formal non-inferiority. Differences between them reflect ablation technology, imaging strategy, study period, operator experience, and heterogeneous definitions of “zero fluoroscopy”. Randomized evidence comparing PFA with thermal ablation shows comparable 12-month efficacy and major complication rates and shorter procedure duration but no consistent reduction in fluoroscopy time—a finding that bears directly on the present topic. Sheath visualization is the decisive enabler: in a randomized trial of 100 patients, left atrial fluoroscopy time was 0 s (IQR 0–0) with a visualizable steerable sheath. In a nonrandomized subgroup analysis of the AdmIRE trial, 12-month freedom from AF was similar across zero- (72.1%), low- (75.4%) and conventional-fluoroscopy (73.6%) strategies. **Conclusions:** Integration of PFA with 3D EAM and intracardiac echocardiography makes a fluoroless workflow practical in experienced hands. The supporting evidence remains predominantly observational; no randomized trial has compared zero-fluoroscopy with fluoroscopy-guided PFA, and the approach described here should be regarded as expert practice rather than a standard of care. Patient safety, not the absolute avoidance of fluoroscopy, must remain the governing principle.

## 1. Introduction

Catheter ablation targeting pulmonary vein isolation (PVI) is the cornerstone of rhythm control for symptomatic atrial fibrillation (AF), supported by multiple randomized controlled trials and endorsed by the 2024 EHRA/HRS/APHRS/LAHRS expert consensus statement on catheter and surgical ablation of AF [1,2]. That document remains the principal contemporary reference for procedural conduct. It addresses the traditional role of fluoroscopy in catheter positioning, transseptal access, pulmonary vein (PV) imaging, and lesion delivery and recognizes the expanding role of electroanatomical mapping and echocardiographic guidance in reducing or eliminating radiation exposure [1].

Conventional procedures rely on fluoroscopy for real-time catheter visualization. Fluoroscopy exposes patients, operators, and laboratory staff to ionizing radiation—a concern amplified in AF ablation, which requires longer fluoroscopy times than simpler electrophysiology procedures and is frequently repeated [3,4].

Pulsed field ablation (PFA) has emerged as a transformative, predominantly non-thermal energy modality that preferentially injures cardiomyocytes through irreversible electroporation, with reduced susceptibility of adjacent structures to injury relative to thermal energy [5,6]. Two PFA-capable catheter systems form the subject of this review, both natively integrated with 3D EAM platforms and therefore well suited to fluoroscopy-free workflows: Sphere-9 (Affera/Medtronic), a 9 mm lattice-tip dual-energy catheter used with the Affera mapping system, and VARIPULSE (Biosense Webster), a variable-loop circular catheter integrated with CARTO 3 [7,8].

In this narrative review, we summarize the harms of radiation exposure, describe the enabling technologies for fluoroless ablation, synthesize the currently available evidence for zero-fluoroscopy ablation and for PFA, and describe a practical approach to performing zero-fluoroscopy AF ablation with these two catheters.

We wish to be explicit about what this review does and does not claim. The evidence supporting PFA as an effective and efficient alternative to thermal ablation derives from trials—ADVENT, SINGLE SHOT CHAMPION, SPHERE Per-AF, AVANT GUARD—that were designed to evaluate energy modality, not to validate a fluoroscopy-free procedural strategy. Conversely, most evidence on zero-fluoroscopy AF ablation derives from observational studies with heterogeneous energy sources, mapping platforms, populations, operator experience, and endpoints. Neither body of evidence validates the other, and combining them does not produce a validated algorithm. The workflow described in Section 9 is therefore presented as an expert- and experience-based approach supported by limited evidence, not as an established standard of care. Each procedural step is labeled in Section 9 according to its evidentiary basis: [C] supported by comparative data, [D] supported by device-specific studies or instructions for use, or [I] institutional practice or expert opinion.

The focus on the Sphere-9 and VARIPULSE catheters is deliberate and narrower than the PFA field as a whole. Both are natively integrated with a three-dimensional electroanatomical mapping platform, are continuously tracked on the map throughout the procedure, and serve as mapping catheters in their own right. They are not, however, the only mapping-integrated PFA catheters in clinical use, and we make no such claim; navigation-enabled pulsed field platforms from other manufacturers are also in clinical use or under evaluation and are considered in Section 10. Other systems in wide use, including the pentaspline and spherical-array platforms, either lack native mapping integration or acquired it later, and fluoroless workflows using them require a separate mapping catheter and a different procedural logic. This review is confined to the Sphere-9 and VARIPULSE systems because these are the two mapping-integrated pulsed field platforms with which the authors have direct procedural experience, and because a step-by-step fluoroless workflow is only worth describing where the authors can vouch for each step.

## 2. Methods

### 2.1. Search Strategy

We searched PubMed/MEDLINE for English-language publications, with no lower date restriction; the final search was executed on 30 June 2026. Embase and the Cochrane Library were not searched, and this is acknowledged as a limitation. The following search string was used:

(“atrial fibrillation”[MeSH] OR “atrial fibrillation”[tiab]) AND (“catheter ablation”[MeSH] OR “catheter ablation”[tiab] OR “pulmonary vein isolation”[tiab]) AND (“zero fluoroscopy”[tiab] OR “fluoroless”[tiab] OR “minimal fluoroscopy”[tiab] OR “near-zero fluoroscopy”[tiab] OR “radiation exposure”[tiab] OR “pulsed field ablation”[tiab] OR “irreversible electroporation”[tiab] OR “Sphere-9”[tiab] OR “Affera”[tiab] OR “lattice tip”[tiab] OR “VARIPULSE”[tiab] OR “variable loop”[tiab] OR “steerable sheath”[tiab] OR “intracardiac echocardiography”[tiab])

Reference lists of all included articles and of the relevant society consensus documents were hand-searched for additional eligible publications.

### 2.2. Eligibility Criteria and Study Selection

Eligible publications were English-language reports of adult human AF ablation reporting procedural or clinical outcomes: randomized controlled trials, prospective or retrospective cohorts, multicenter registries, systematic reviews and meta-analyses, and society guideline or consensus documents. Studies confined to pediatric populations, ablation of arrhythmias other than AF (except where reporting radiation-exposure data of direct relevance), case reports of fewer than 10 patients, and non-English publications were excluded.

Conference abstracts, preprints, and non-peer-reviewed manufacturer-sponsored reports were not used to support any numerical statement. Peer-reviewed articles published online ahead of print were eligible and are cited with their digital object identifier.

Two authors (R.K. and A.C.) searched and compiled candidate literature independently and without a shared protocol; results were pooled and duplicates removed, and the senior author (K.K.) adjudicated inclusion. Generative artificial intelligence assistance (Claude, Anthropic) was used to help identify candidate literature and to draft and revise the manuscript text. All citations were subsequently verified by the authors against the primary publication or an authoritative bibliographic index, and statements that could not be verified were removed. The authors take full responsibility for the accuracy and integrity of all content.

As this is a narrative rather than a systematic review, no formal risk-of-bias instrument was applied, no certainty-of-evidence grading was undertaken, and no quantitative pooling was performed. Reporting follows general recommendations for narrative reviews rather than PRISMA. Quantitative statements reproduced in this review are taken directly from the primary publications and were not re-analyzed.

### 2.3. Definitions

Terminology in this literature is inconsistent, which materially affects the comparability of reported results. We use the following definitions throughout. Zero fluoroscopy denotes a procedure completed with a total fluoroscopy time of 0.0 min and no activation of the radiation source at any point. Near-zero fluoroscopy denotes minimal but non-zero exposure, and we report the actual threshold used by each source rather than adopting a single cut-off, since published thresholds range from <0.5 min to ≤5 min. Low fluoroscopy denotes exposure above the near-zero range but substantially below institutional convention, and in this review refers specifically to the >0 to ≤5 min stratum used in the AdmIRE fluoroscopy analysis. Crossover denotes conversion from an intended zero-fluoroscopy procedure to fluoroscopy use at any point, for any reason. Where a cited study uses a different definition, this is stated at the point of citation.

We also distinguish fluoroscopy time from radiation dose throughout. These are not interchangeable: dose depends on the frame rate, collimation, magnification, patient body habitus and equipment generation as well as on exposure duration, and a reduction in fluoroscopy time does not imply a proportionate reduction in dose.

## 3. Detrimental Effects of Radiation Exposure

Radiation exposure during catheter ablation carries both deterministic effects (skin erythema, cataract) and stochastic effects (malignancy, heritable defects), the latter conventionally modeled under a linear no-threshold assumption [9,10]. Among the most heavily exposed cardiac catheterization laboratory staff, cumulative occupational dose confers a non-negligible lifetime attributable risk of cancer: applying the BEIR VII model to individual dosimetric histories, the median lifetime risk of fatal or non-fatal cancer was 1 in 192 (IQR 1 in 137 to 1 in 370) [11].

Case series of interventional physicians have reported a striking left-sided predominance of brain and neck tumors. Among 31 affected physicians, information on tumor laterality was available in 26, of whom 22 (85%) were left-sided, one midline and three right-sided—a distribution that departs sharply from the symmetrical distribution seen in the general population and is consistent with asymmetric cranial exposure, the operator standing to the patient’s right with the radiation source to the left [12]. Dedicated dosimetry has confirmed the mechanism: in the BRAIN study, radiation exposure to the operator’s cranium was significantly greater on the left and center than on the right and was attenuated by a non-lead barium sulphate/bismuth oxide cap [13]. Radiation-associated posterior subcapsular lens opacities have been documented in 52% of interventional cardiologists and 45% of nurses, compared with 9% of unexposed controls [14].

The orthopedic burden of personal protective equipment is a distinct and under-recognized harm. Approximately half of interventional cardiologists report at least one orthopedic injury, most commonly of the cervical or lumbar spine, with prevalence rising with age and cumulative caseload [15,16]. In a contemporary Heart Rhythm Society membership survey, orthopedic injuries were reported by 41% of respondents, of whom 68% required physical therapy and 23% required surgery; premature cataract before the age of 50 was reported by 3.7% [17]. Eliminating fluoroscopy removes the rationale for wearing lead at all, and this ergonomic benefit is arguably as consequential for workforce longevity as the oncological one.

Patient-level factors compound radiation risk. Body mass index is a stronger determinant of delivered dose than total fluoroscopy time (r = 0.74 vs. r = 0.37), and mean effective doses rise stepwise across body-habitus categories: 15.2 ± 7.8 mSv in normal-weight, 26.7 ± 11.6 mSv in overweight, and 39.0 ± 15.2 mSv in obese patients; during one hour of fluoroscopy an obese patient receives approximately 3.2 times the dose-area product of a normal-weight patient [18]. A meta-analysis confirmed that patients with overweight or obesity undergoing radiofrequency (RF) ablation experience longer procedures and receive larger radiation doses without a corresponding excess of procedural complications [19]. When pre-procedural computed tomography (CT) is included in the calculation, obese patients receive a cumulative effective dose approximately 75% higher than normal-weight patients [20]. A fluoroless strategy is therefore of particular value in patients with obesity, in whom conventional dose-reduction measures are least effective.

Two qualifications are important. First, fluoroscopy time and radiation dose must not be conflated, as noted in Section 2.3; studies reporting only time may substantially misrepresent the exposure actually delivered. Second, contemporary ultra-low-dose fluoroscopy systems—using reduced frame rates, aggressive collimation, spectral filtration and advanced image processing—can reduce dose by an order of magnitude relative to older equipment while retaining fluoroscopic guidance. Much of the comparative literature reviewed here predates the widespread availability of such systems, and the marginal dose benefit of a fluoroless strategy over a modern ultra-low-dose protocol is smaller than the historical comparisons imply. The residual advantages of a fluoroless approach are the elimination of dose rather than its reduction and the removal of the requirement to wear leaded protection.

The ALARA (“as low as reasonably achievable”) principle, endorsed by all major electrophysiology societies, holds that no threshold of ionizing radiation can be considered entirely safe [3,9]. The 2023 HRS expert consensus statement on the management of arrhythmias during pregnancy recommends zero-fluoroscopy techniques when ablation is necessary during pregnancy [21]. Pediatric patients are particularly vulnerable owing to greater radiosensitivity and a longer remaining lifespan over which stochastic effects may be expressed [10].

## 4. Enabling Technologies for Fluoroless AF Ablation

### 4.1. Three-Dimensional Electroanatomical Mapping

Contemporary 3D EAM systems—principally CARTO 3 (Biosense Webster, Irvine, CA, USA), EnSite X (Abbott, St. Paul, MN, USA), and the Affera mapping system (Medtronic, Mounds View, MN, USA)—provide real-time catheter visualization using magnetic and impedance-based tracking and are used routinely by the large majority of AF ablation operators [3]. The multicenter “Go for Zero Fluoroscopy” project (25 centers, 1793 procedures) demonstrated that 3D mapping significantly reduces both fluoroscopy time and dose across all categories of electrophysiology procedure (*p* < 0.001) [22]. The 2026 ACC/AHA/HRS advanced training statement identifies competency in 3D mapping as a core trainee skill [23].

### 4.2. Intracardiac Echocardiography

Intracardiac echocardiography (ICE) provides real-time imaging of cardiac anatomy, guides transseptal puncture, assists in assessing catheter–tissue relationships, and enables early detection of complications [3,24]. The largest available synthesis pooled 44 AF ablation studies comprising 482,043 patients and found that ICE guidance was associated with lower odds of any complication (OR 0.69, 95% CI 0.53–0.89), cardiac tamponade (OR 0.58, 95% CI 0.53–0.62), and mortality (OR 0.21, 95% CI 0.16–0.27), together with shorter procedure and fluoroscopy times, lower radiation exposure, higher odds of first-pass PVI (OR 1.62, 95% CI 1.09–2.41), and lower odds of repeat ablation (OR 0.65, 95% CI 0.59–0.72); atrial tachyarrhythmia recurrence did not differ (OR 0.92, 95% CI 0.79–1.06) [25]. An earlier meta-analysis reached directionally similar conclusions on efficiency and safety [26]. These are observational data and residual confounding by center expertise cannot be excluded, but the consistency of effect across a very large denominator is notable.

ICE-guided transseptal puncture without fluoroscopy, using right atrial electroanatomical mapping to localize the fossa ovalis, has been reported with high success and low complication rates [27]. Transesophageal echocardiography has also been used to guide fluoroless procedures where ICE is unavailable [28]. The CARTOSOUND FAM module applies a deep-learning algorithm to ICE images to reconstruct left atrial anatomy automatically on the CARTO 3 map, eliminating manual contouring and the need for pre-procedural CT or magnetic resonance imaging (MRI) [29].

### 4.3. Visualizable Steerable Sheaths

Sheath visualization is central to the workflow proposed here and merits detailed consideration, because the sheath—not the ablation catheter—is what usually accounts for residual fluoroscopy once mapping and ICE are in place.

A systematic review and meta-analysis of steerable versus non-steerable sheaths for AF ablation established the general case: steerable sheaths improve catheter stability and procedural efficiency without a signal of increased complications [30]. An earlier pooled analysis of 10 studies and 967 patients reported a higher rate of freedom from atrial arrhythmia with steerable sheaths (RR 1.19, 95% CI 1.09–1.29, *p* < 0.001) and shorter procedure times (MD −10.6 min), with comparable complication rates [31].

The more directly relevant question is whether making the sheath visible on the mapping system permits a radiation-free left atrial workflow. In a single-center randomized trial, 100 consecutive patients undergoing PVI were allocated 50:50 to a visualizable or a standard non-visualizable steerable sheath. Acute ablation success was 100% in both arms, and first-pass isolation rates were similar (92% vs. 89%, *p* = 0.88). Left atrial procedure time was shorter with the visualizable sheath (53.1 [41.3; 73.1] vs. 59.5 [47.6; 74.1] min, *p* = 0.04). Critically, left atrial fluoroscopy time was 0 s (IQR 0–0) versus 17.5 s (IQR 5.5–69.3) (*p* < 0.01), and left atrial fluoroscopy dose was 0 mGy (IQR 0–0.27) versus 0.74 mGy (IQR 0.16–2.34) (*p* < 0.01). That is, no further fluoroscopy was required during the left atrial portion of the procedure in the visualizable-sheath group. Total procedure time, total fluoroscopy time, and total fluoroscopy dose did not differ between arms, because the residual radiation was concentrated in the access and transseptal phases [32].

This randomized finding is the strongest single piece of evidence underpinning the workflow described in Section 9. Once transseptal access has been achieved, direct visualization of the steerable sheath on the electroanatomical map is sufficient to complete the left atrial component of the procedure without radiation, providing considerably firmer support than the observational sheath literature alone. Consistent with this, a single-center study of a visualizable steerable sheath reported reduced fluoroscopy time (3.4 vs. 5.8 min, *p* = 0.003) and dose (10.0 vs. 18.5 mGy, *p* = 0.015) compared with non-visualizable sheaths [33], and the multicenter INSIGHT cohort reproduced reductions in fluoroscopy time and dose in real-world practice [34]. One qualification must be stated explicitly, because it constrains how this evidence may be used. All three of these studies [32,33,34] were performed on the CARTO 3 platform with a steerable sheath rendered directly on that mapping system. They therefore establish the value of sheath visualization on CARTO 3 and cannot be extrapolated to other electroanatomical mapping platforms, on which sheath rendering is implemented differently. Where the workflow described in Section 9 relies on sheath localization outside the CARTO 3 environment, it does so on the platform-specific grounds set out in Section 9.5 and not on the strength of these trials.

### 4.4. Contact and Tissue Proximity Feedback

Catheters that provide objective feedback on catheter–tissue interaction reduce operator reliance on fluoroscopy for inferring contact. In zero-fluoroscopy AF ablation cohorts, contact-force sensing has been associated with shorter procedure times when combined with EAM and ICE [35]. In PFA, analogous information is supplied by impedance-based indices—tissue proximity indication (TPI) on CARTO 3 for VARIPULSE and local impedance for Sphere-9—described in Section 7 and Section 8.

## 5. Evidence for Zero-Fluoroscopy AF Ablation

Four meta-analyses now address this question, and their consistency is more informative than any single estimate. An important caveat applies to all four: each pools comparative studies that were not designed as non-inferiority trials and did not pre-specify a non-inferiority margin. They therefore demonstrate an absence of detected difference, which is not the same as demonstrated non-inferiority. We use the language of comparability rather than non-inferiority throughout, and readers should interpret the pooled estimates accordingly.

Kanitsoraphan et al. (16 studies, 6052 patients) found no difference between zero-fluoroscopy and conventional approaches in acute success (OR 1.10), recurrence-free survival (OR 1.08), or complications (OR 0.72), with a crossover rate from zero-fluoroscopy to fluoroscopy of only 1.26% [36]. Huang et al. (15 studies, 2228 patients) reported comparable 12-month AF recurrence (OR 1.34, non-significant) with significantly shorter procedure time (WMD −14.6 min) and fluoroscopy time (WMD −8.8 min) [37].

Debreceni et al. restricted their analysis specifically to AF ablation and pooled seven studies comprising 1593 patients (726 zero-fluoroscopy, 867 non-zero-fluoroscopy; one randomized trial and six observational studies). The zero-fluoroscopy approach was feasible in 95.1% of patients. Compared with a non-zero-fluoroscopy strategy it significantly reduced procedure time (MD −9.11 min, 95% CI −12.93 to −5.30; *p* < 0.01), fluoroscopy time (MD −5.21 min, 95% CI −5.51 to −4.91; *p* < 0.01), and fluoroscopy dose (MD −3.96 mGy, 95% CI −4.27 to −3.64; *p* < 0.01), without penalty in acute or long-term success or in complication rates [38]. Because this analysis is confined to AF and explicitly reports feasibility, it is arguably the most directly applicable of the four to the present topic.

A 2026 meta-analysis of 12 studies comprising 1998 patients (two randomized trials and ten observational cohorts; 1098 zero-fluoroscopy and 900 conventional-fluoroscopy procedures) found no significant difference in 12-month freedom from atrial arrhythmia recurrence (OR 0.98, 95% CI 0.62–1.57, *p* = 0.74) or in overall procedural complications (OR 0.73, 95% CI 0.35–1.55, *p* = 0.42), alongside a significant reduction in radiation exposure [39].

### 5.1. Why Do the Pooled Estimates Differ?

The point estimates across these analyses are directionally concordant but not numerically identical. The differences are explicable rather than contradictory, and five sources of heterogeneity account for most of the variation.

The first factor is the ablation technology. The earlier analyses are dominated by point-by-point RF ablation, whereas more recent cohorts include cryoballoon, high-power short-duration RF, and PFA. Single-shot and wide-footprint technologies shorten left atrial dwell time and therefore compress the absolute differences a fluoroless strategy can generate.

The second factor is the imaging strategy. Studies differ in whether ICE was mandatory, optional, or unavailable. ICE-based workflows achieve zero fluoroscopy through direct anatomical visualization; EAM-only workflows depend on electroanatomical landmarking and sheath visualization and tend to report higher crossover.

The third factor is the study period and operator experience. Procedures performed early in an institution’s fluoroless transition carry the learning curve; later cohorts do not. Pooling across a decade therefore mixes learning-phase with steady-state performance.

The fourth factor is the definition of “zero fluoroscopy”. Definitions range from a strict 0.0 min of radiation, through thresholds such as <0.5 min, to “no fluoroscopy after transseptal access”. Analyses that admit near-zero cohorts will attenuate apparent differences relative to those requiring absolute zero.

The fifth factor is the comparator heterogeneity. The control arm ranges from high-fluoroscopy conventional practice to already-optimized low-dose protocols, which materially changes the achievable reduction.

### 5.2. Single-Center and Registry Data

Key non-randomized studies reinforce the pooled findings. Torma et al. demonstrated shorter procedure times with zero fluoroscopy (59.6 vs. 74.6 min, *p* < 0.0001) with 100% acute PVI [40]. The SHORT LOOK registry (450 patients) achieved near-zero fluoroscopy (median 26 s) without ICE, with 82.6% freedom from AF at 12 months, demonstrating that an EAM-only route is viable where ICE is unavailable [41]. Liu et al. combined a zero-fluoroscopy strategy with high-power short-duration ablation and reported 85.6% one-year freedom from arrhythmia [42]. A simplified ICE-guided fluoroless workflow has also been shown to extend to repeat procedures [43].

## 6. Pulsed Field Ablation: Clinical Evidence

PFA delivers microsecond-scale, high-voltage electrical pulses that create nanopores in cell membranes, inducing irreversible electroporation. Its defining characteristic is relative tissue selectivity: myocardium has a lower field threshold for irreversible injury than the esophagus, phrenic nerve, and vascular structures, so that at clinically used field strengths these adjacent tissues show reduced susceptibility to injury and a lower observed incidence of collateral damage than with thermal energy [5,6,44]. This is a difference of degree rather than of kind, and PFA-specific injury remains possible, as Section 6.1 describes.

The ADVENT trial (607 patients) demonstrated non-inferiority of PFA to thermal ablation for both efficacy (73.3% vs. 71.3%) and safety (2.1% vs. 1.5% serious adverse events), with superior preservation of PV dimensions [5]. The ADVENT-LTO analysis showed favorable outcomes maintained at four years, with significantly fewer repeat ablations (10.4% vs. 17.7%, *p* = 0.04) [45]. The SINGLE SHOT CHAMPION trial (210 patients) demonstrated non-inferiority of PFA to cryoballoon ablation, with a lower recurrence rate (37.1% vs. 50.7%) assessed by continuous rhythm monitoring [46]. The AVANT GUARD trial extended the evidence to persistent AF as a first-line strategy: 310 patients were randomized 2:1 to PFA with a pentaspline catheter or to antiarrhythmic drug therapy, all with an insertable cardiac monitor. Treatment success at 12 months was 56% with PFA versus 30% with drug therapy (HR for treatment failure 0.46, 95% CI 0.33–0.65, *p* < 0.001), with device- or procedure-related serious adverse events in 5.1% of each group [47]. The comparator in that trial was pharmacological, not thermal ablation, and the result should be read as supporting PFA as an initial rhythm-control strategy rather than as a comparison between energy sources.

The MANIFEST-US registry confirmed safety at scale: 41,968 patients treated at 102 US centers by more than 500 operators between February 2024 and July 2025, with a major adverse event rate of 0.63% and no reported esophageal fistula, PV stenosis, or persistent phrenic nerve palsy [48]. These figures apply specifically to the pentaspline catheter and should not be assumed to transfer unchanged to other PFA platforms; the earlier MANIFEST-17K study reported a consistent profile for the same device [49]. Meta-analyses report lower AF recurrence with PFA than with RF (OR 0.68) and cryoballoon ablation (RR 0.81), with shorter procedure times [50,51]. Beyond the two catheters that are the subject of this review, the PULSAR pivotal trial of a spherical multielectrode array catheter in 183 patients with paroxysmal AF reported a 12-month effectiveness of 77.8% against a performance goal of 50% (*p* < 0.001), with durable PVI in 95% of veins after a single application, illustrating the pace at which the PFA field is diversifying [52].

### 6.1. Reconciling the Comparative Syntheses

Several systematic reviews and meta-analyses of PFA versus thermal ablation have appeared in rapid succession, and they are not fully concordant. A sequential list of citations would obscure the disagreement; the differences are instructive and are set out here.

Analyses restricted to randomized evidence are more conservative. Pooling the available randomized trials in paroxysmal AF, Ali et al. found comparable procedural efficacy and major complication rates between PFA and thermal or non-PFA ablation, together with significantly shorter procedure duration but—critically for the present review—no significant difference in fluoroscopy time, with substantial heterogeneity across trials [53]. Krishan et al., also restricting to randomized trials in paroxysmal AF, reached concordant conclusions on 12-month efficacy and procedural efficiency [54], as did a further randomized-only synthesis that characterized PFA as a comparable and more efficient alternative while emphasizing the influence of operator experience and rhythm-monitoring strategy on apparent success rates [55].

Analyses that incorporate observational data tend to report more favorable estimates for PFA, including lower arrhythmia recurrence against radiofrequency and cryoballoon comparators and fewer specific collateral injuries [50,51]. An updated mixed-design synthesis incorporating both randomized and observational studies with time-to-event analysis reached similar conclusions [56], and a technology-specific meta-analysis restricted to multielectrode catheter-based pulsed electric field versus cryoballoon ablation likewise favored PFA within that narrower comparison [57]—a reminder that pooled estimates are sensitive to which comparator and which PFA platform are admitted. These estimates carry greater clinical and methodological heterogeneity, are susceptible to confounding by center and operator selection, and have in some cases identified safety signals—including hemolysis and coronary vasospasm—that the randomized datasets were underpowered to detect.

The discrepancies are attributable to design (randomized-only versus mixed), AF phenotype (paroxysmal versus persistent versus mixed cohorts), PFA platform (pentaspline, lattice-tip, variable-loop and spherical-array systems are pooled together in several analyses despite differing markedly in lesion geometry and application count), comparator (radiofrequency, cryoballoon, or both), rhythm-monitoring intensity (intermittent monitoring systematically overestimates freedom from arrhythmia relative to continuous monitoring), follow-up duration, and the certainty of evidence attaching to each.

Two conclusions follow, and the second is the one that matters for this review. First, PFA is best characterized at present as comparably effective and safe relative to thermal ablation, with a consistent procedural-efficiency advantage; claims of superiority rest largely on observational data. Second, and directly relevant here, the randomized evidence does not demonstrate that PFA per se reduces fluoroscopy exposure [53]. Any reduction in radiation achieved in the workflows described in this review is attributable to the mapping, imaging and sheath-visualization strategy rather than to the energy source. Evidence addressing fluoroscopy-free PFA specifically [58] answers a different question from the energy-comparison literature and is considered separately in Section 11. The two should not be conflated, and evidence supporting PFA as an alternative to thermal ablation should not be read as validating a fluoroscopy-free workflow.

### 6.2. PFA-Specific Safety Considerations

PFA introduces its own safety profile rather than simply removing thermal risk. Recognized signals include coronary arterial spasm during ablation near the coronary arteries, intravascular hemolysis—which, in procedures with high application counts, has rarely precipitated acute kidney injury requiring temporary hemodialysis—and silent cerebral emboli detected on post-procedural imaging [5,6]. The 2026 EHRA/HRS/APHRS/LAHRS/CHRS scientific statement provides comprehensive guidance on biophysics, platform taxonomy, clinical evidence, workflow, and training and identifies vasospasm, hemolysis, and cerebrovascular events as the priority signals requiring harmonized surveillance, together with post-ablation atrial tachyarrhythmia as an outcome warranting systematic reporting [6]. These considerations bear directly on a fluoroless workflow, since none of them is detected by fluoroscopy, whereas pericardial effusion and catheter malposition—the complications fluoroscopy might reasonably be expected to reveal—are detected earlier and more reliably by ICE [25].

## 7. The Sphere-9 Catheter and the Affera Mapping System

### 7.1. Nomenclature

Terminology in this field is inconsistent, and the components of the Sphere-9 platform are frequently conflated in published reports. For clarity, the following convention is used throughout this review, and no other term is substituted.

### 7.2. Device Description

Sphere-9 is an 8-F bidirectional deflectable catheter bearing a 9 mm compressible nitinol lattice tip carrying nine mini-electrodes with embedded thermocouples, used with the Affera mapping system for high-density mapping, navigation, and lesion delivery. Local impedance at each mini-electrode provides electrode-level proximity information, and each mini-electrode carries a temperature sensor permitting tailored power titration. Its distinguishing feature is dual-energy capability: the operator can toggle between temperature-controlled RF and pulsed field energy from the same catheter, without catheter exchange, via a foot pedal [7].

### 7.3. Clinical Evidence

The SPHERE Per-AF trial (NCT05120193) randomized 420 patients with persistent AF 1:1 to the dual-energy lattice-tip system or to conventional contact-force-sensing RF ablation with CARTO 3. The trial met its primary non-inferiority endpoint for effectiveness (73.8% vs. 65.8% freedom from AF at 12 months) with a primary safety event rate of 1.4% versus 1.0% in the control arm, no PV stenosis, no esophageal events, and improved procedural efficiency [7]. A pre-specified analysis of linear lesions within the trial showed shorter ablation and energy-application times for every lesion type with the investigational catheter (all *p* < 0.0001), with left atrial roof or posterior wall isolation performed in 95.8%, cavotricuspid isthmus (CTI) line in 55.2%, and mitral line in 35.8% of the investigational arm [59]. An operator learning-curve analysis found that efficiency gains were achieved rapidly despite the majority of operators being new to the system [60].

Fluoroscopy use with this platform is low but not zero. An indirect treatment comparison against a contemporaneous pentaspline PFA trial found fluoroscopy time to be approximately 14 min shorter with the Affera platform after adjustment (−14.4 min, 95% CI −16.2 to −12.5; *p* < 0.01), with no significant difference in skin-to-skin or PVI times [61]. In a comparative appraisal of fluoroscopy exposure across PFA platforms, average fluoroscopy time with the focal 9 mm lattice-tip system was reported as 4.4 ± 3.1 min, with a range extending to 14.1 min [62]. No dedicated prospective series of fully zero-fluoroscopy Sphere-9 ablation has been published.

This asymmetry with the VARIPULSE evidence base (Section 8.2) should be stated openly rather than glossed, but it does not, in our view, preclude a fluoroless Sphere-9 workflow, for the following reason. What renders an AF ablation fluoroless is not the ablation catheter but the enabling stack that precedes it: ultrasound-guided venous access, ICE-guided navigation of the right atrium, electroanatomical tagging of the fossa ovalis and adjacent landmarks, ICE- and map-guided transseptal puncture, and direct localization of the steerable sheath by the converging methods described in Step 4. Each of these elements is identical in the two workflows described in Section 9, and each is supported by the evidence reviewed in Section 4 and Section 5—including, on the CARTO 3, the randomized demonstration that left atrial fluoroscopy time falls to zero once the sheath is visualizable [32]. The ablation catheter is the final element rather than the enabling one, and both catheters are continuously tracked within their respective mapping platforms. The Sphere-9 fluoroless workflow presented here is therefore a reasoned extension of an evidenced foundation rather than a validated protocol, and we present it as such.

## 8. The VARIPULSE Catheter

### 8.1. Device Description

VARIPULSE is a variable-loop circular catheter with an adjustable loop diameter of 25–35 mm and bidirectional deflection. It is fully integrated with CARTO 3, providing real-time non-fluoroscopic navigation, lesion tagging, and tissue proximity indication (TPI). Pulsed field energy is delivered by the TRUPULSE generator [8,63].

### 8.2. Clinical Evidence

The AdmIRE pivotal trial (277 patients, 30 US centers) reported a median procedure time of 81 min, a 12-month primary effectiveness of 74.6%, and a primary adverse event rate of 2.9%, with low-fluoroscopy exposure; more than a quarter of procedures were completed without fluoroscopy (first-quartile fluoroscopy time 0.0 min) [8]. The earlier inspIRE study reported 12-month outcomes for the same catheter with 3D mapping integration [64].

A dedicated retrospective analysis of AdmIRE addressed the fluoroscopy question directly and is the most informative dataset available on this point. Index ablations performed by 49 operators across 30 centers were segmented into zero-fluoroscopy (0 min), low-fluoroscopy (>0 to ≤5 min), and conventional-fluoroscopy (>5 min) subgroups. Twelve-month freedom from AF was 73.5% overall with similar estimates across the three subgroups: 72.1% with zero fluoroscopy, 75.4% with low fluoroscopy, and 73.6% with conventional fluoroscopy. Complication rates trended lower with zero- and low-fluoroscopy approaches than with conventional fluoroscopy, although the differences were not statistically significant. Notably, conventional-fluoroscopy operators tended to be more experienced, which would, if anything, have biased the comparison against the fluoroless groups [65]. Subgroup allocation was neither randomized nor pre-specified, the comparison was not designed as an equivalence or non-inferiority analysis, and no equivalence margin was defined. These data should therefore be read as showing similar estimates, or as failing to detect a difference between subgroups, rather than as establishing equivalence; residual confounding by operator experience, center and patient selection cannot be excluded.

Dedicated fluoroless series have followed. Chan et al. reported the first dedicated zero-fluoroscopy VARIPULSE series: 34 consecutive patients, all completed without fluoroscopy, a median procedure time of 40.5 min, no major complications, the use of two sheaths, and no pre-procedural CT or MRI [66]. Borlich et al. described a near-zero-fluoroscopy workflow with the same catheter in an initial cohort of 35 patients [62]. Teumer et al. described a “mapping-on-the-fly” optimization, reducing procedure time to 68 min [67]. Fink et al. confirmed 100% acute PVI in 45 patients (mean 66.3 min) [68]. Andria et al. demonstrated feasibility for non-PV targets including posterior wall isolation, roof lines, and CTI ablation [69]. The largest dedicated series to date is a prospective study of 121 consecutive patients undergoing PVI with or without linear ablation using this catheter, in which a fully standardized fluoroscopy-free workflow was feasible in 98%. That workflow was guided by CARTO 3 with transesophageal rather than intracardiac echocardiography, which the authors advance on cost-efficiency grounds and which demonstrates that ICE is not an absolute prerequisite for a fluoroless approach; the comparatively lower success of cavotricuspid isthmus ablation in that cohort, however, suggests that ICE retains value for non-pulmonary-vein targets [70]. Comparable near-zero-fluoroscopy workflows have been described for other circular multielectrode PFA catheters [71].

Artificial-intelligence-assisted ICE reconstruction has been evaluated with this catheter. In a retrospective multicenter study of 157 consecutive patients (61% paroxysmal, 39% persistent) across four Italian centers, left atrial reconstruction using the CARTOSOUND FAM module (n = 64) was compared with conventional electroanatomical reconstruction (n = 93) with propensity-score matching, demonstrating the feasibility and procedural impact of AI-assisted ICE mapping during PFA [72].

In a single-center comparison of the three PFA systems available in Japan (pentaspline, VARIPULSE, and PulseSelect; 75 patients), total lesion areas were similar but lesion patterns differed; the pentaspline catheter had the shortest procedure time, whereas VARIPULSE was associated with the lowest radiation exposure of the three [73]. This is consistent with the mapping-system integration of the variable-loop catheter but derives from a small single-center cohort and should not be over-interpreted.

## 9. Institutional Fluoroless Workflow

The following describes the standard workflow at our institution. It has not been prospectively evaluated against an alternative strategy and is presented as an expert- and experience-based approach, not as a validated or generalizable algorithm. Each step carries a label indicating its evidentiary basis: [C], supported by comparative data, [D], supported by device-specific studies or instructions for use, and [I], institutional practice or expert opinion. Steps 1 to 5 are common to both catheter systems; Section 9.3 and Section 9.4 describe the system-specific components.

### 9.1. Patient Selection and Pre-Procedural Assessment

The first step is patient selection [I]. We do not regard a fluoroless strategy as appropriate for every patient. Procedures anticipated to be anatomically complex, patients with prior cardiac surgery or congenital abnormality of the interatrial septum, those with prior device leads crossing the region of interest, and repeat procedures after prior transseptal puncture with septal scarring are all situations in which we plan for fluoroscopy to be used if required rather than committing to a fluoroless approach at the outset. Operator and laboratory experience are prerequisites: the published learning curve for zero-fluoroscopy PFA plateaus after approximately 114 cases [74], and centers early in that curve should expect a higher crossover rate.

**Figure 1:**
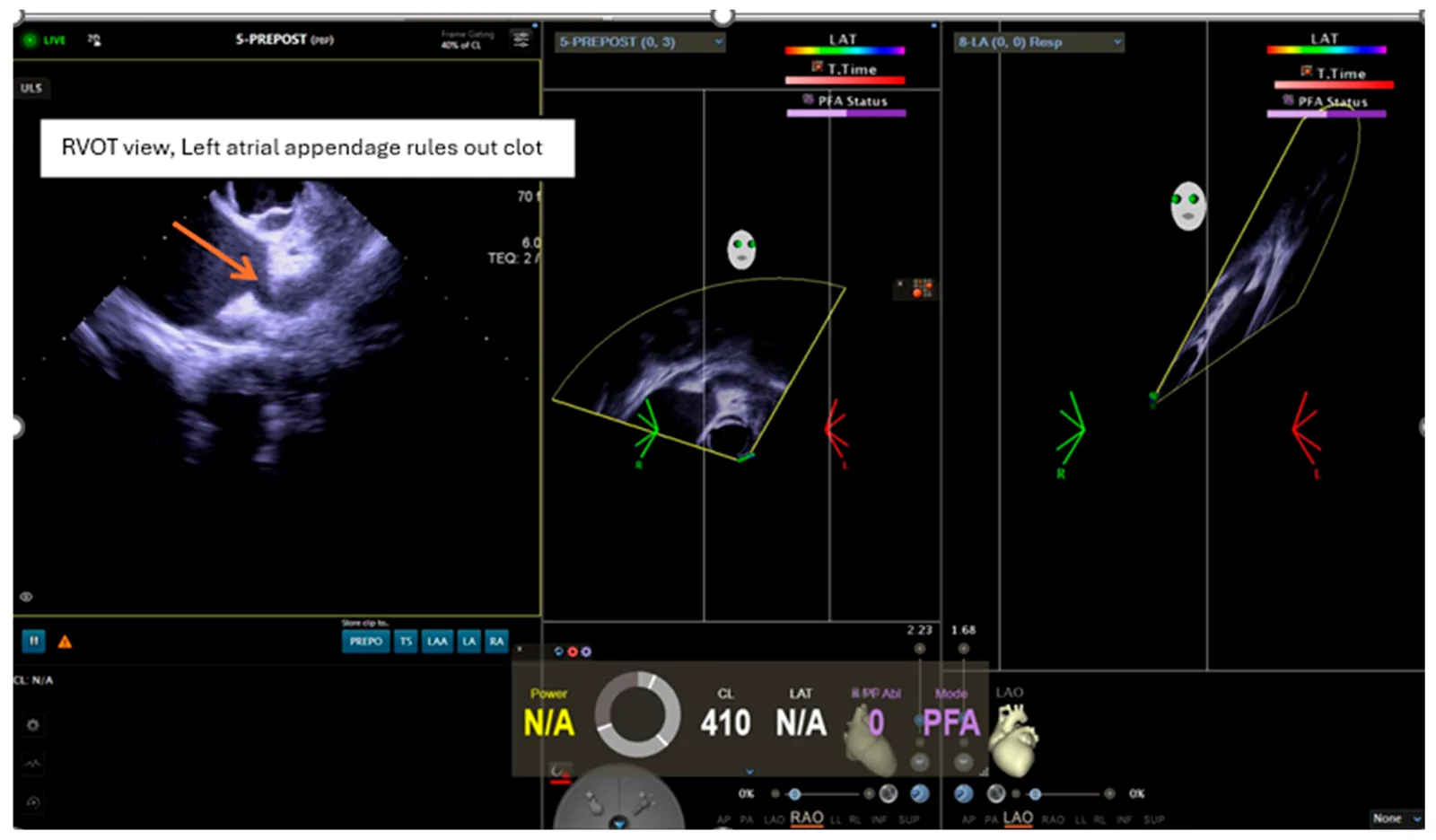
Intracardiac echocardiography (ICE) assessment of the left atrial appendage (LAA) from the right ventricular outflow tract (RVOT) view, ruling out left atrial appendage thrombus prior to ablation. The RVOT view is obtained when the standard right atrial ICE window is inadequate. This step is performed at the start of every fluoroless procedure as part of the pre-ablation safety assessment. RVOT, right ventricular outflow tract; LAA, left atrial appendage.

For exclusion of left atrial appendage thrombus, all patients undergo pre-procedural imaging to exclude LAA thrombus. Our practice is to perform this with ICE at the start of the procedure, imaging the LAA from the right atrium and, when the right atrial view is inadequate, advancing the ICE catheter into the right ventricular outflow tract to obtain a dedicated LAA window (Figure 1). In our experience, this view is of comparable diagnostic quality to transesophageal echocardiography (TEE) for this purpose. We state plainly, however, that this is an operator- and image-quality-dependent assessment and that ICE from right-sided chambers should not be presented as universally sufficient. Reported sensitivity of ICE for LAA thrombus varies with catheter position, imaging window, appendage morphology and operator experience, and a non-diagnostic or equivocal ICE examination must be resolved rather than assumed negative.

We proceed to TEE or cardiac computed tomography, accepting the associated logistical burden or radiation exposure, in the following circumstances: an inadequate or equivocal ICE window; sub-therapeutic or interrupted anticoagulation in the preceding weeks; a high thromboembolic risk profile with spontaneous echo contrast or reduced LAA emptying velocities; known prior LAA thrombus; complex or multilobed appendage anatomy; and any situation in which the operator is not confident that thrombus has been excluded. The governing principle is that a fluoroless strategy must never become a reason to accept a less certain answer to the thrombus question.

The next step is pre-procedural cross-sectional imaging [I]. We do not routinely perform pre-procedural cardiac CT or MRI for anatomical definition, relying instead on intraprocedural ICE and fast anatomical mapping. CT is obtained when unusual pulmonary venous anatomy is suspected, when prior imaging is discordant, or when it is required for another indication.

### 9.2. Bailout Criteria and the Governing Principle

A fluoroless strategy requires explicit, pre-agreed criteria for abandoning it [I]. Fluoroscopy is immediately available in the laboratory for every case, the team is briefed that its use is expected rather than exceptional in the circumstances below, and no operator is discouraged from requesting it.

We use fluoroscopy promptly when: the position of a catheter, wire or sheath is uncertain on the electroanatomical map or cannot be confirmed on ICE; resistance is encountered during advancement of any wire, dilator, sheath, or catheter; the transseptal apparatus cannot be confidently localized relative to the fossa ovalis; anatomy is complex, distorted, or discordant with the acquired map; a complication is suspected, including pericardial effusion, tamponade, air embolism, or vascular injury; equipment malfunction or loss of catheter tracking occurs; or the procedure is taking materially longer than anticipated without clear progress. Crossover to fluoroscopy is recorded as such rather than treated as a failure of technique.

The reported crossover rate from zero-fluoroscopy to conventional fluoroscopy is low at 1.26% [36], and feasibility in the most rigorous AF-specific pooled analysis was 95.1% [38]—that is, approximately one procedure in twenty was not completed without radiation. These figures should be read as reassurance that crossover is uncommon, not as a target to be minimized. Patient safety, rather than the absolute avoidance of fluoroscopy, must remain the governing principle of any fluoroless program, and a laboratory that regards crossover as a failure has misunderstood the objective.

### 9.3. Steps Common to Both Systems

**Step 1: Vascular access, anticoagulation, and catheter preparation [I/D].** All procedures are performed under general anesthesia. Ultrasound-guided right femoral venous access is obtained to minimize vascular complications and to support a fully fluoroless workflow. Our standard access strategy comprises the Baylis VersaCross transseptal system, an 11-Fr Pinnacle sheath, and a VIZIGO bidirectional steerable sheath. The mapping and ablation catheters are advanced through the VIZIGO sheath. An ICE catheter (SOUNDSTAR for CARTOSOUND integration or ACUSON AcuNav) is introduced through the 11-Fr sheath and a decapolar coronary sinus (CS) catheter through the Baylis sheath. Routine esophageal temperature monitoring is not used during PFA at our institution. Anticoagulation is managed as follows. All procedures are performed on uninterrupted oral anticoagulation for 4 weeks, with the morning dose taken on the day of the procedure. An initial intravenous heparin bolus is administered immediately after venous access and before transseptal puncture, dosed by weight, with the activated clotting time (ACT) first measured 10 min after the bolus and thereafter every 20 to 30 min. Further boluses or a continuous infusion is given to maintain an ACT of 350–400 s throughout the left atrial portion of the procedure. Protamine is administered at the end of the case to reverse residual heparinization before sheath removal. Sheath allocation and the sequence of transseptal equipment are as follows, since these determine whether the workflow is reproducible. Three venous accesses are obtained in the right femoral vein. The Baylis VersaCross transseptal system is used for the transseptal puncture and, before that, carries the decapolar coronary sinus catheter. The 11-Fr Pinnacle sheath carries the ICE catheter for the duration of the case. The VIZIGO bidirectional steerable sheath carries the mapping catheter during right atrial mapping, then the transseptal dilator and radiofrequency wire during the puncture, and thereafter the ablation catheter. The order of assembly is: ICE catheter first, then the coronary sinus catheter, then right atrial mapping through the VIZIGO sheath, then exchange of the mapping catheter for the transseptal apparatus within the same sheath, then transseptal puncture, and then introduction of the ablation catheter through the VIZIGO sheath into the left atrium. Irrigation and generator settings follow the manufacturer instructions for use for each system and are not modified at our institution [D]. For the VARIPULSE catheter, pulsed field energy is delivered by the TRUPULSE generator at its fixed output; for the Sphere-9 catheter, pulsed field applications are delivered by the HexaPulse generator and temperature-controlled radiofrequency by the HexaGen generator with HexaFlow irrigation, with power and temperature targets set per the instructions for use. We do not use investigational or off-label parameter settings.

**Step 2: ICE catheter advancement and intracardiac anatomical assessment [C/I].** The ICE catheter is advanced from the inferior vena cava (IVC) into the right atrium (RA) under continuous ultrasound visualization, with careful rotation and deflection to maintain a longitudinal view of the venous lumen and minimize vessel-wall contact. Sequential clockwise rotation from the “home view” (RA, tricuspid valve, aortic root, right ventricular outflow tract) demonstrates the fossa ovalis, mitral valve, left atrial appendage (LAA), left PVs, posterior left atrial (LA) wall, and right PVs. The LAA is assessed for thrombus; where additional visualization is required, a right ventricular or right ventricular outflow tract imaging window is obtained. The superior vena cava (SVC)-RA junction, IVC-RA junction, tricuspid annulus, CS ostium, and fossa ovalis are systematically identified as landmarks for fluoroless navigation and transseptal access.

**Figure 2:**
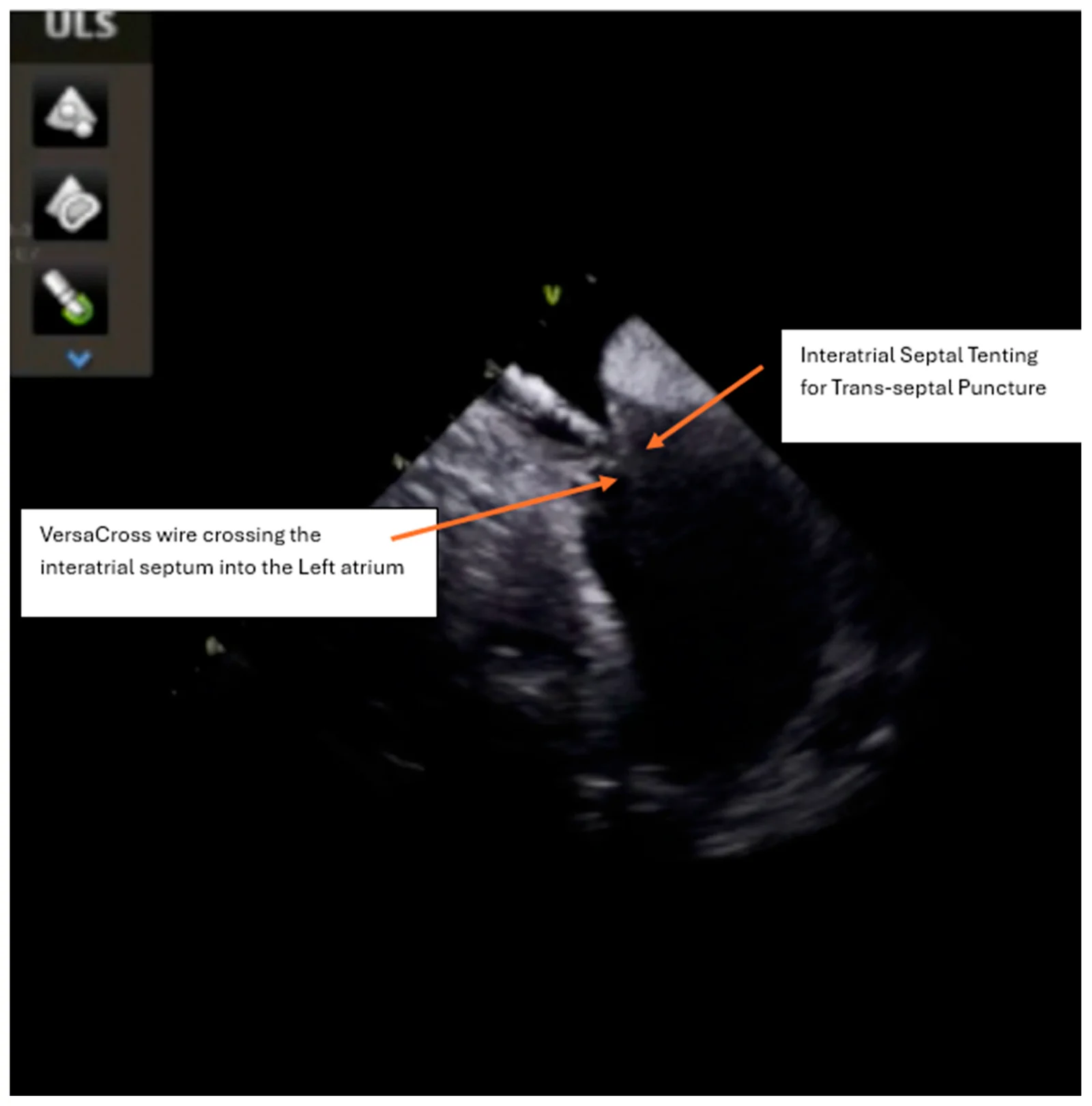
Fluoroless transseptal puncture under intracardiac echocardiography (ICE) and CARTO 3 guidance. Septal tenting at the mid-posterior, slightly inferior portion of the interatrial septum (arrow) with the radiofrequency-enabled VersaCross wire advanced across the interatrial septum into the left atrium (arrowhead).

**Step 3: Coronary sinus catheter placement [I].** The decapolar CS catheter is advanced into the coronary sinus under combined ICE and 3D EAM guidance without fluoroscopy. The position is confirmed by characteristic intracardiac electrograms and by visualization on the mapping system (Figure 2). The CS catheter thereafter serves as a stable intracardiac reference and is used for pacing maneuvers, assessment of atrial activation, and confirmation of conduction block.

**Step 4: Transseptal puncture—sheath positioning [C].** The mapping or ablation catheter is advanced into the SVC under 3D EAM guidance, and the VIZIGO sheath is then advanced over it into the SVC. The basis for sheath localization differs between the two platforms and should not be conflated. On CARTO 3 the VIZIGO bidirectional steerable sheath is displayed directly on the electroanatomical map, and comparative studies have shown that sheath visualization reduces fluoroscopy time and dose during atrial fibrillation ablation [33,34]; these data were generated exclusively on CARTO 3 and are not transferable to other mapping platforms. On the Affera/Prism platform, localization is achieved by hybrid magnetic and calibrated-impedance (“synergy”) navigation, which, per the manufacturer’s system documentation, also provides visualization of non-sensor-based diagnostic catheters such as a coronary sinus catheter [75]. That documentation refers to diagnostic catheters and not to sheaths; in our own procedures the VIZIGO sheath can likewise be appreciated on the Affera map in relation to the Sphere-9 catheter (Section 9.5), which we report as a procedural observation rather than as a documented platform feature. No comparative data on sheath visualization exist for this platform, and we therefore do not rely on the map alone. In practice the sheath is localized on both systems by three converging methods: direct intracardiac echocardiographic visualization of the sheath tip as it is advanced through the inferior vena cava, the right atrium and, subsequently, across the interatrial septum; the position of the catheter over which the sheath is being delivered, which is always advanced first under electroanatomical guidance; and the sheath position as rendered on the mapping system. The sheath is never advanced blindly—it is advanced only over a catheter or wire whose position has been confirmed both on intracardiac echocardiography and on the electroanatomical map— and fluoroscopy is used without hesitation if the sheath position is at any point uncertain. The mapping catheter is removed, and the transseptal dilator and radiofrequency-enabled VersaCross wire are introduced. The sheath–dilator–wire assembly is positioned in the SVC and withdrawn slowly toward the interatrial septum, with position and orientation monitored by simultaneous ICE imaging and 3D EAM. This dual-modality approach permits precise fluoroless localization of the transseptal apparatus relative to the fossa ovalis and is the technique for which zero-fluoroscopy transseptal access has been most consistently reported [27].

**Step 5: Transseptal puncture—fossa ovalis engagement and left atrial crossing [C].** The assembly is withdrawn until engagement and tenting of the fossa ovalis are directly visualized on ICE, with simultaneous confirmation on the electroanatomical map. Our preferred puncture site is the mid-posterior and slightly inferior fossa ovalis, individualized to LA anatomy and the anticipated lesion set. Once tenting and site are confirmed, radiofrequency energy is applied through the transseptal wire, and the wire is advanced across the septum (Figure 2). Entry into the LA is confirmed by direct ICE visualization of the pigtail configuration of the wire within the LA cavity. The wire is advanced into a left pulmonary vein, and the dilator and sheath are advanced over it into the LA under continuous ICE and mapping guidance; the dilator and wire are then removed. From this point onward the procedure is conducted entirely without fluoroscopy. On the CARTO 3/VARIPULSE platform this rests on direct visualization of the VIZIGO sheath on the electroanatomical map, the approach evaluated in the randomized sheath-visualization trial discussed in Section 4.3, in which left atrial fluoroscopy time was 0 s [32]; on the Affera/Sphere-9 platform, for which no comparable trial exists, it rests on the combination of intracardiac echocardiography, catheter-first sheath advancement and map rendering described in Step 4.

### 9.4. VARIPULSE Workflow

**Figure 3:**
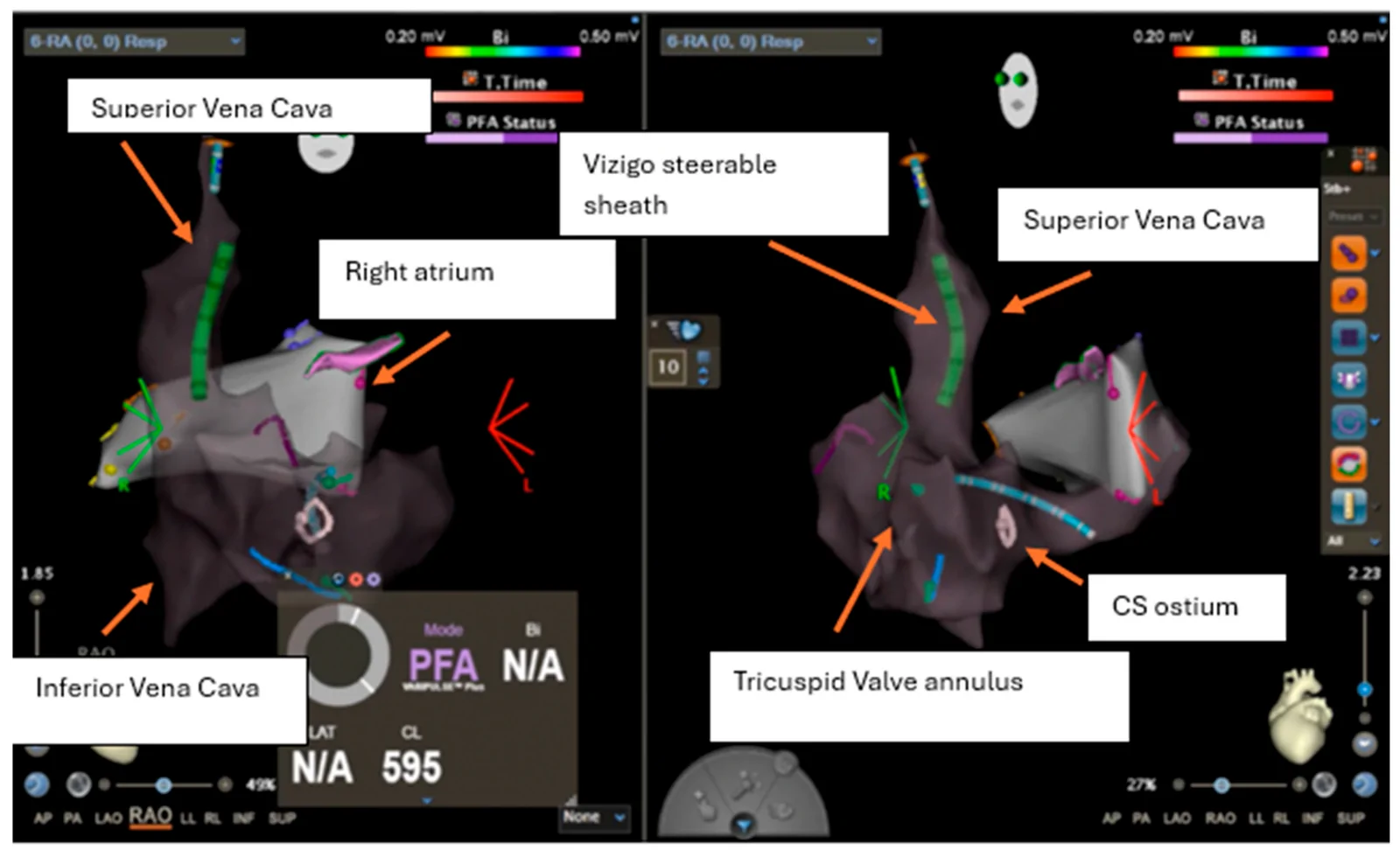
Right atrial electroanatomical map created with the OCTARAY catheter on CARTO 3 prior to left atrial mapping. Tagged landmarks: superior vena cava–right atrial junction, inferior vena cava–right atrial junction, tricuspid annulus, and coronary sinus ostium. The VIZIGO steerable sheath (arrow) is directly visualized on the map, enabling fluoroless navigation.

**Step 6: Right atrial mapping and landmark identification [D/I].** Before detailed LA mapping, a limited right atrial map is created on CARTO 3 using an OCTARAY high-density mapping catheter. The SVC-RA junction, IVC-RA junction, tricuspid annulus, CS ostium, and fossa ovalis are identified and electronically tagged (Figure 3). These tags facilitate fluoroless navigation and provide spatial orientation during advancement and manipulation of the VIZIGO sheath. The CS catheter is visualized on the map as an additional stable anatomical and electrical reference.

**Figure 4:**
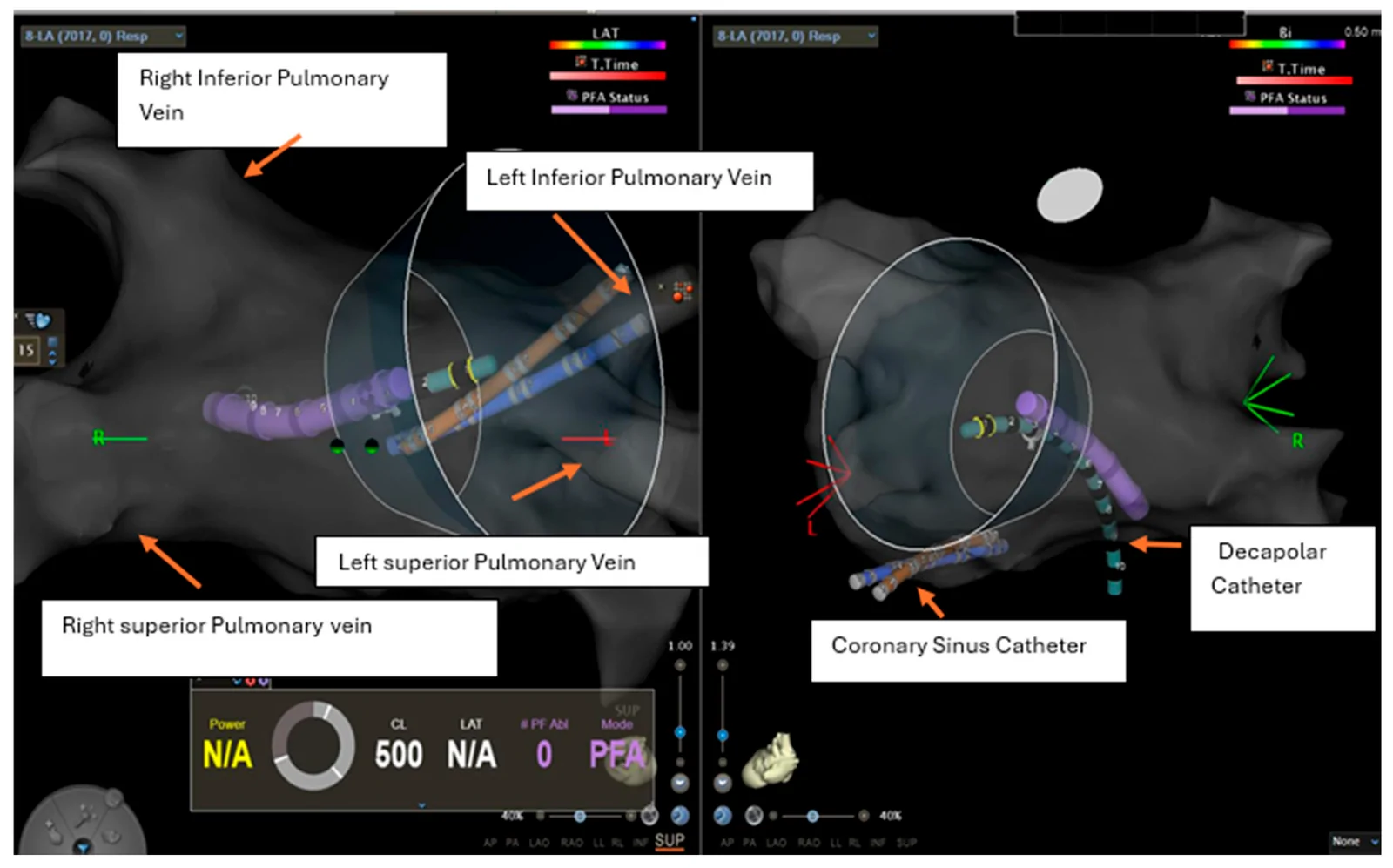
Three-dimensional left atrial reconstruction with catheters in situ on the CARTO 3/VARIPULSE system (posteroanterior view, left panel; superior/roof view, right panel). Key structures are labeled: decapolar catheter, coronary sinus catheter, left superior pulmonary vein, left inferior pulmonary vein, right superior pulmonary vein, and right inferior pulmonary vein. This anatomical reconstruction is created by combining SOUNDSTAR ICE catheter imaging via the CARTOSOUND FAM module with fast anatomical mapping using the OCTARAY catheter and provides the navigational foundation for all subsequent fluoroless left atrial catheter manipulation and ablation.

**Step 7: Three-dimensional left atrial reconstruction [D].** Following transseptal access, detailed reconstruction of the LA and PVs is performed on CARTO 3, combining anatomical information from the SOUNDSTAR ICE catheter via CARTOSOUND integration with fast anatomical mapping using the OCTARAY catheter. The reconstruction delineates the LA chamber, all four PV ostia and antra, the LAA, the left PV-LAA ridge, and the posterior LA wall (Figure 4). The previously acquired right atrial geometry remains visible, and the tagged fossa ovalis and CS catheter provide references for safe return to the RA.

Pre-procedural cardiac CT or MRI is not routinely performed in our workflow, consistent with published fluoroless series [66].

**Figure 5:**
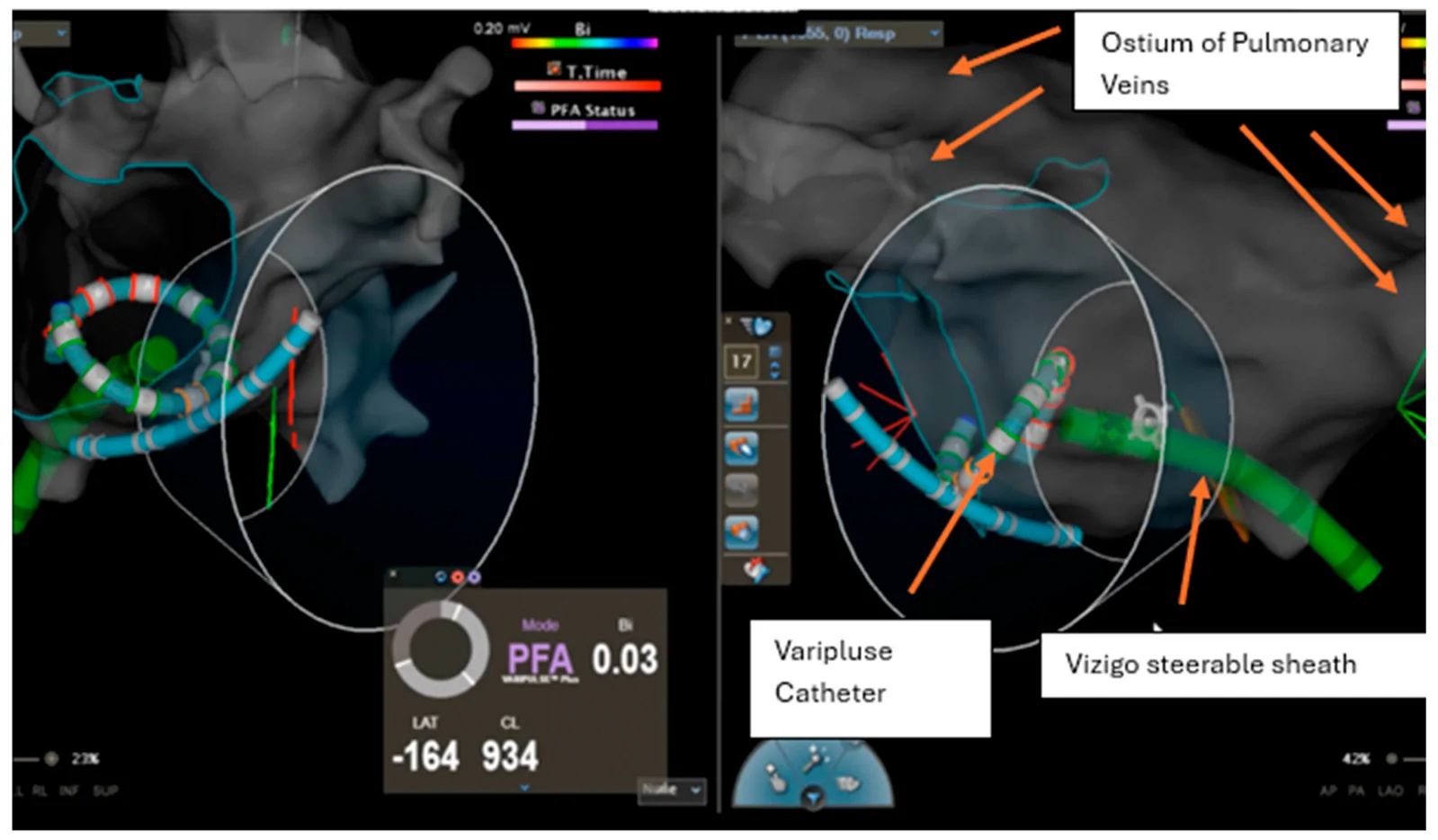
Introduction of the VARIPULSE variable-loop circular catheter into the left atrium via the VIZIGO sheath under CARTO 3 and ICE guidance. The catheter is unsheathed in the mid-left atrial cavity, with the loop diameter adjusted between 25 and 35 mm for optimal pulmonary vein engagement. Labels identify the ostium of pulmonary veins, VARIPULSE catheter, and VIZIGO steerable sheath.

**Step 8: Catheter introduction and tissue proximity assessment [D].** The transseptal wire and dilator are removed, and the VARIPULSE catheter is introduced through the VIZIGO sheath into the LA under combined CARTO 3 and ICE guidance. The catheter is unsheathed within the mid-LA cavity to minimize inadvertent contact with adjacent structures, and the loop diameter is adjusted between 25 and 35 mm according to the target vein (Figure 5). TPI is activated during advancement and positioning. TPI uses unipolar impedance measurement to detect electrode proximity to tissue (<0.2 mm indicating contact) and is distinct from the bipolar impedance reported by the TRUPULSE generator; the two should not be conflated. Two visualization modes are available: binary (default; white bands indicate proximity) and continuous (sphere size reflects the tissue proximity ratio, larger indicating better contact). ICE is used concurrently to confirm catheter orientation relative to the PV ostium and antrum [63].

**Figure 6:**
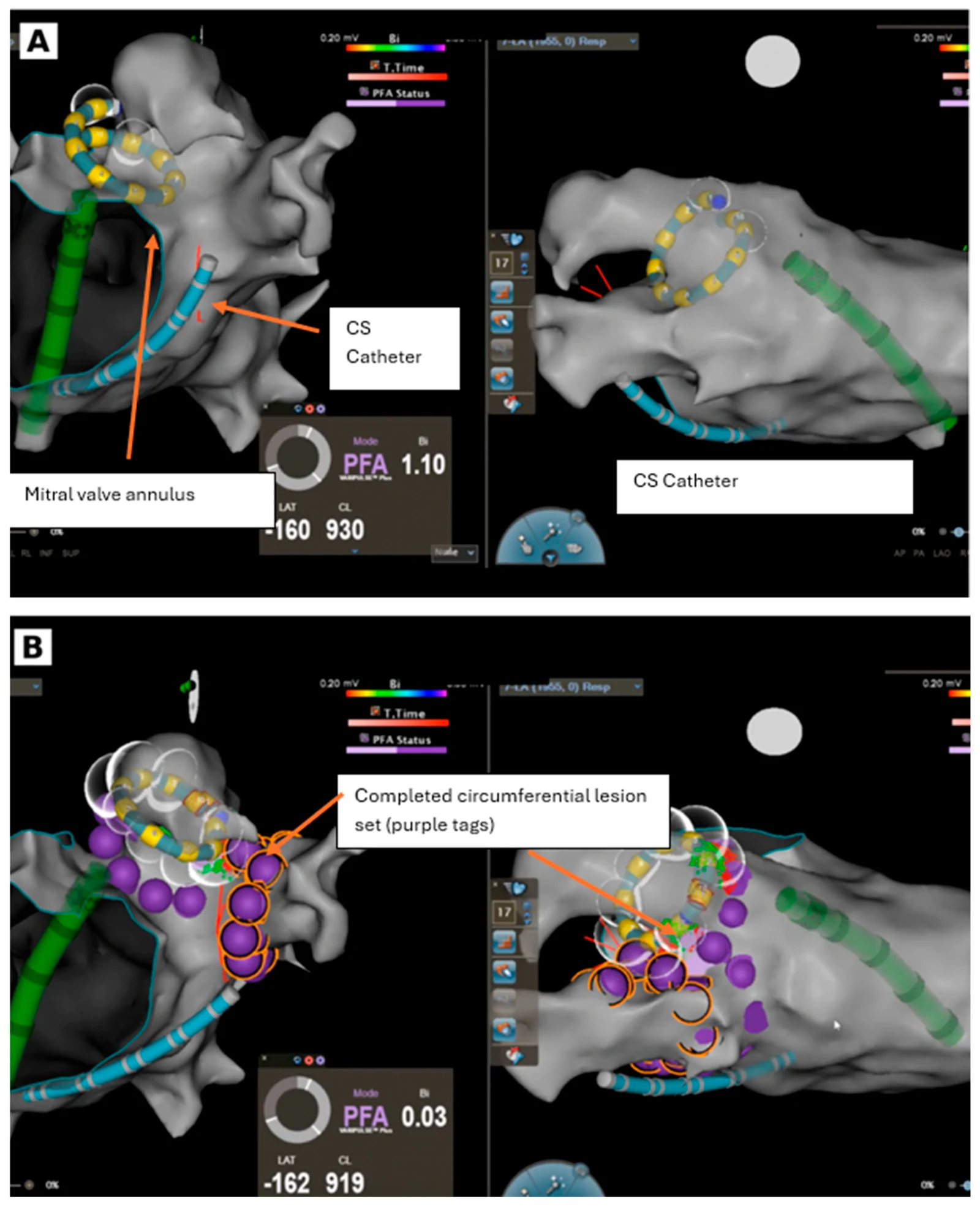
Pulsed field ablation delivery with the VARIPULSE catheter using the TRUPULSE generator. (**A**) Circumferential applications around the pulmonary vein ostium and antrum with systematic catheter rotation, with CS catheter and mitral valve annulus labeled. (**B**) Completed circumferential lesion set (purple tags) marking all applied ablation points around the pulmonary veins, ensuring contiguous coverage. The arrow marks the junction between electrodes 1 and 10, where spatial gaps are most likely to occur.

**Step 9: Pulmonary vein mapping and pulsed field ablation [D/I].** Baseline PV electrograms and local atrial activation are assessed before ablation, and catheter position and tissue proximity are evaluated using TPI, the electroanatomical map, and real-time ICE. Pulsed field energy is delivered with the TRUPULSE generator circumferentially around each PV, with systematic catheter rotation and repositioning between applications (Figure 6) and with particular attention to avoiding spatial gaps between electrodes 1 and 10 and to achieving complete circumferential coverage. A minimum of 16 PFA applications per procedure is generally delivered as part of our standard PVI strategy, using both ostial and antral positions as appropriate to achieve wide-area circumferential isolation. Catheter position is reassessed before each application. An application is accepted when the tissue proximity indication demonstrates electrode–tissue proximity across the intended arc, the catheter is stable on the map through the application, and the generator reports delivery without an error condition. An application is repeated when the proximity indication shows a gap across part of the loop, when catheter displacement is observed during delivery, when the generator aborts, or when post-application mapping demonstrates residual conduction in the corresponding segment.

**Figure 7:**
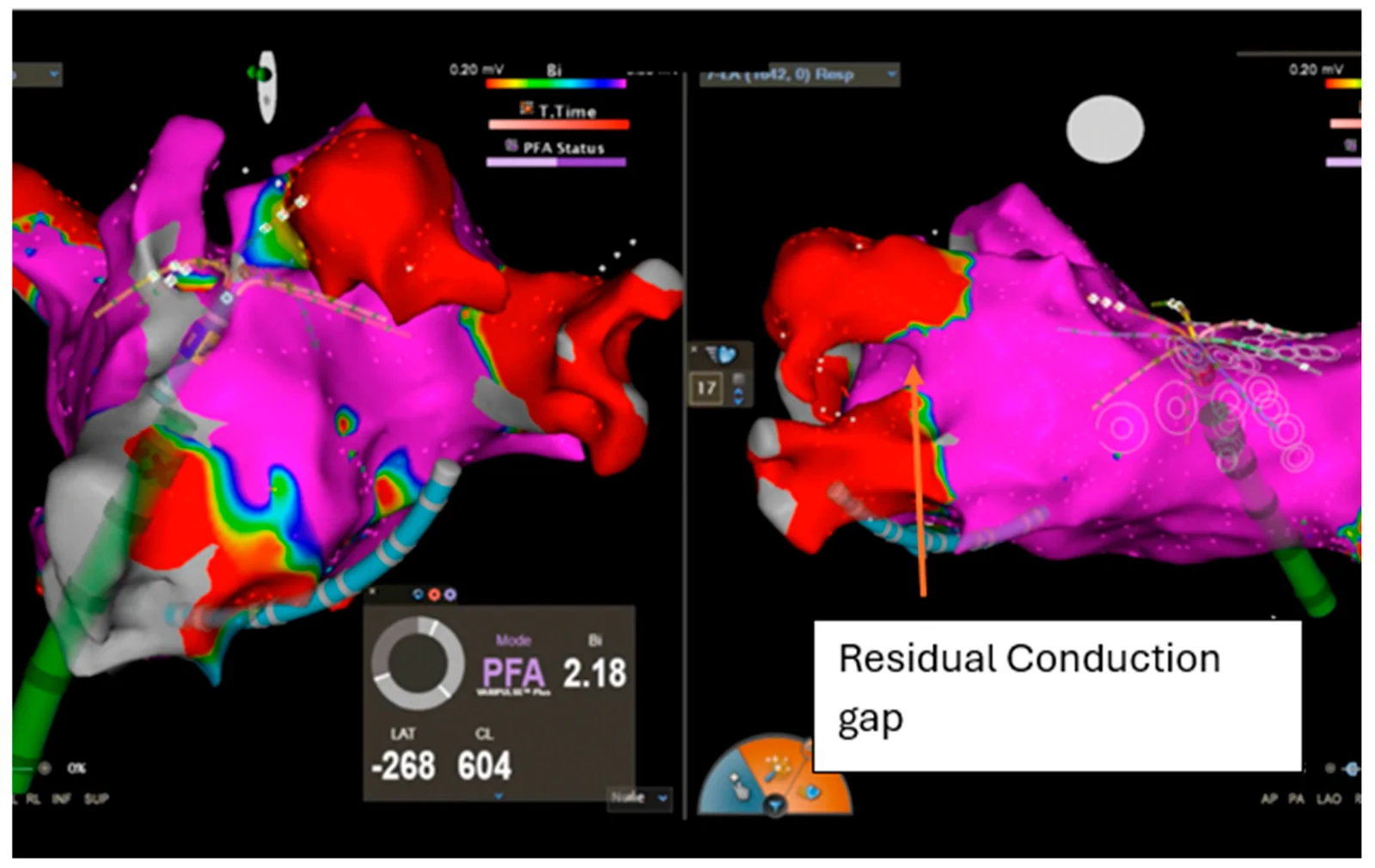
Post-ablation remapping of the pulmonary vein antra and ostia with the OCTARAY high-density mapping catheter, confirming entrance and exit block. Purple indicates ablated low-voltage tissue; arrow marks a residual conduction gap subsequently targeted with an additional application. ICE verifies the anatomical position of the mapping catheter relative to the pulmonary vein ostium.

**Step 10: Confirmation of pulmonary vein isolation [D].** All PVs are systematically reassessed for electrical isolation, and entrance and exit block are confirmed in each vein. The OCTARAY catheter is used to remap the PV antra and ostia, with ICE confirming the anatomical position of the mapping catheter relative to the PV ostium and surrounding LA structures (Figure 7). Residual conduction gaps identified on the map are targeted with additional PFA applications, and remapping is repeated until bidirectional block is demonstrated. We describe this as acute bidirectional block rather than durable isolation: no routine waiting period, adenosine or isoproterenol provocation, or protocol-mandated chronic remapping is performed, and acute block at the end of the procedure does not establish durability.

**Step 11: Additional lesion sets [I].** Additional lesions are performed when clinically indicated according to the presenting arrhythmia, atrial substrate, and procedural strategy; posterior wall isolation may be performed with PFA in selected patients. At our institution, the VARIPULSE catheter is not routinely used for mitral isthmus or CTI ablation; where linear ablation of these regions is indicated, an alternative catheter and energy source are used. We note that others have reported feasibility of non-PV targets with this catheter, and our practice reflects institutional preference rather than a limitation of the device [69].

**Step 12: Post-ablation assessment, anticoagulation reversal, and vascular closure [I].** The VARIPULSE catheter is withdrawn into the sheath and the catheter–sheath system returned to the RA under mapping and ICE guidance. A comprehensive post-ablation ICE examination is performed to exclude pericardial effusion, intracardiac thrombus, and significant valvular injury. Protamine is administered to reverse heparinization where appropriate. After sheath removal, femoral venous hemostasis is achieved with a vascular closure device (Perclose ProGlide or MynxGrip, according to operator preference and access characteristics). The patient is awakened and transferred to the recovery area.

### 9.5. Sphere-9 Workflow

**Figure 8:**
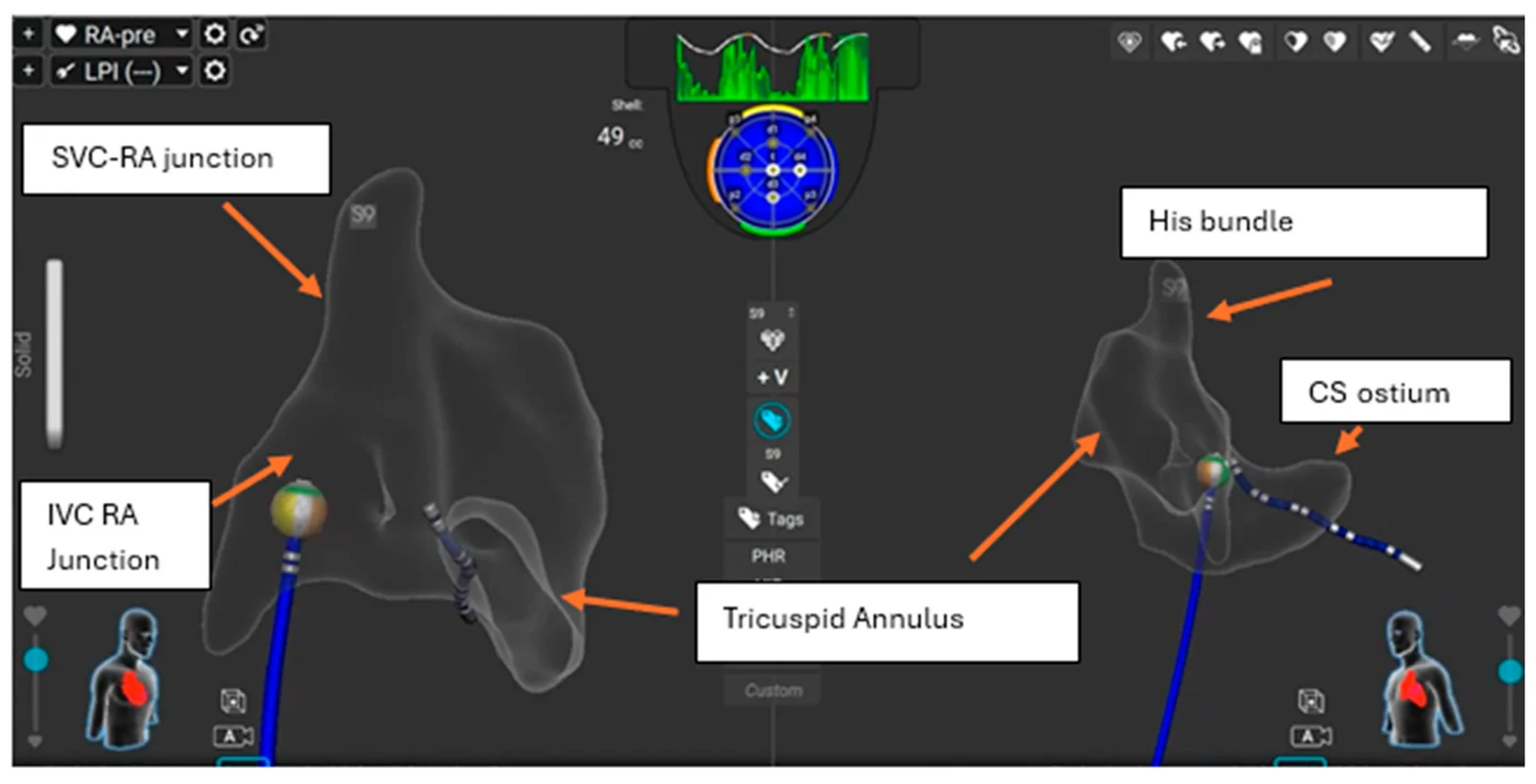
Right atrial electroanatomical map created with the Sphere-9 lattice-tip catheter on the Affera mapping system prior to transseptal puncture. Tagged landmarks include the SVC-RA junction, IVC RA junction, His bundle, tricuspid annulus, and CS ostium.

**Figure 9:**
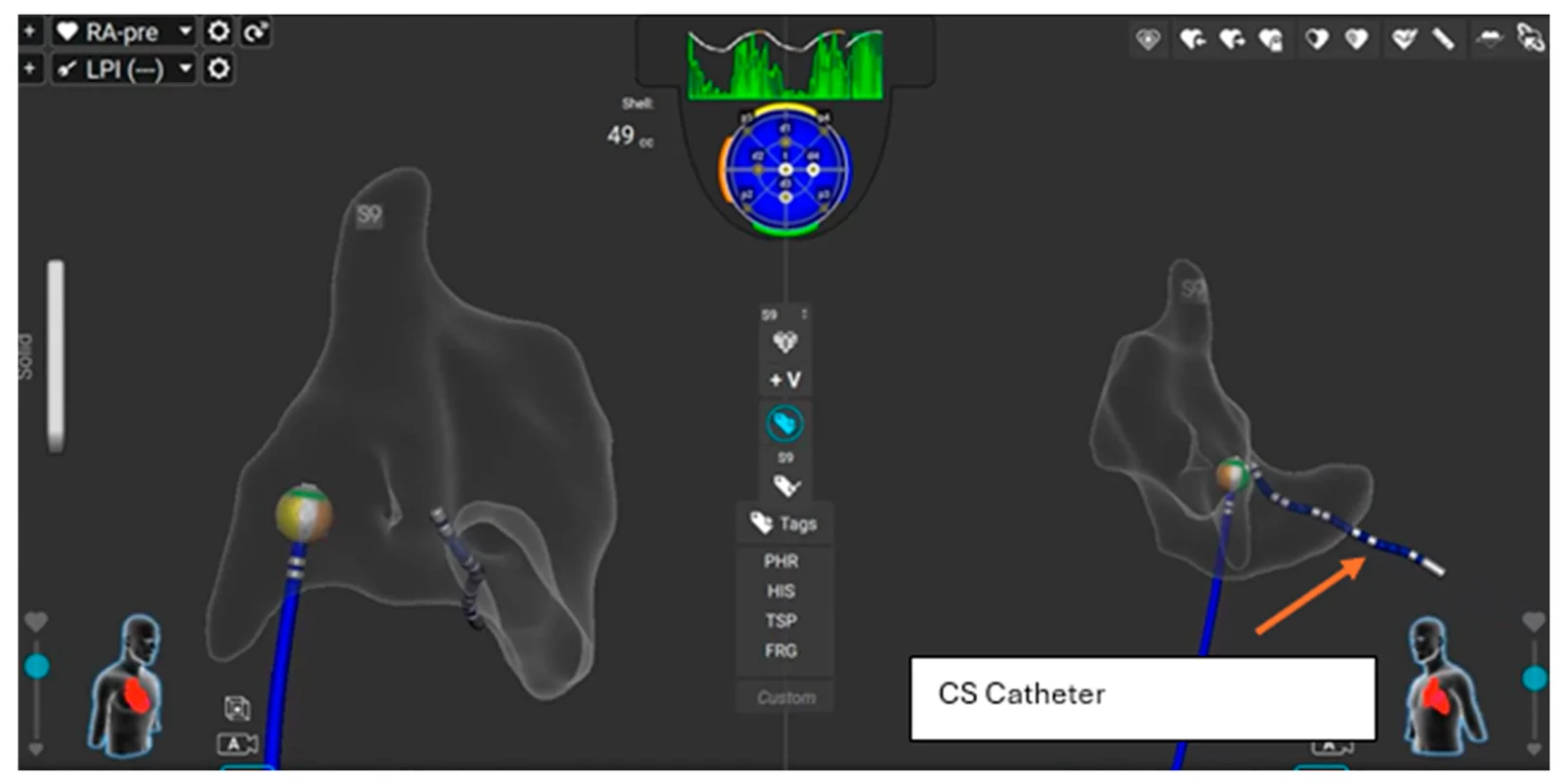
Coronary sinus catheter placement under combined Sphere-9 mapping and ICE guidance on the Affera mapping system. The coronary sinus catheter (CS Catheter, arrow) serves as a stable anatomical and electrical reference throughout the procedure.

**Figure 10:**
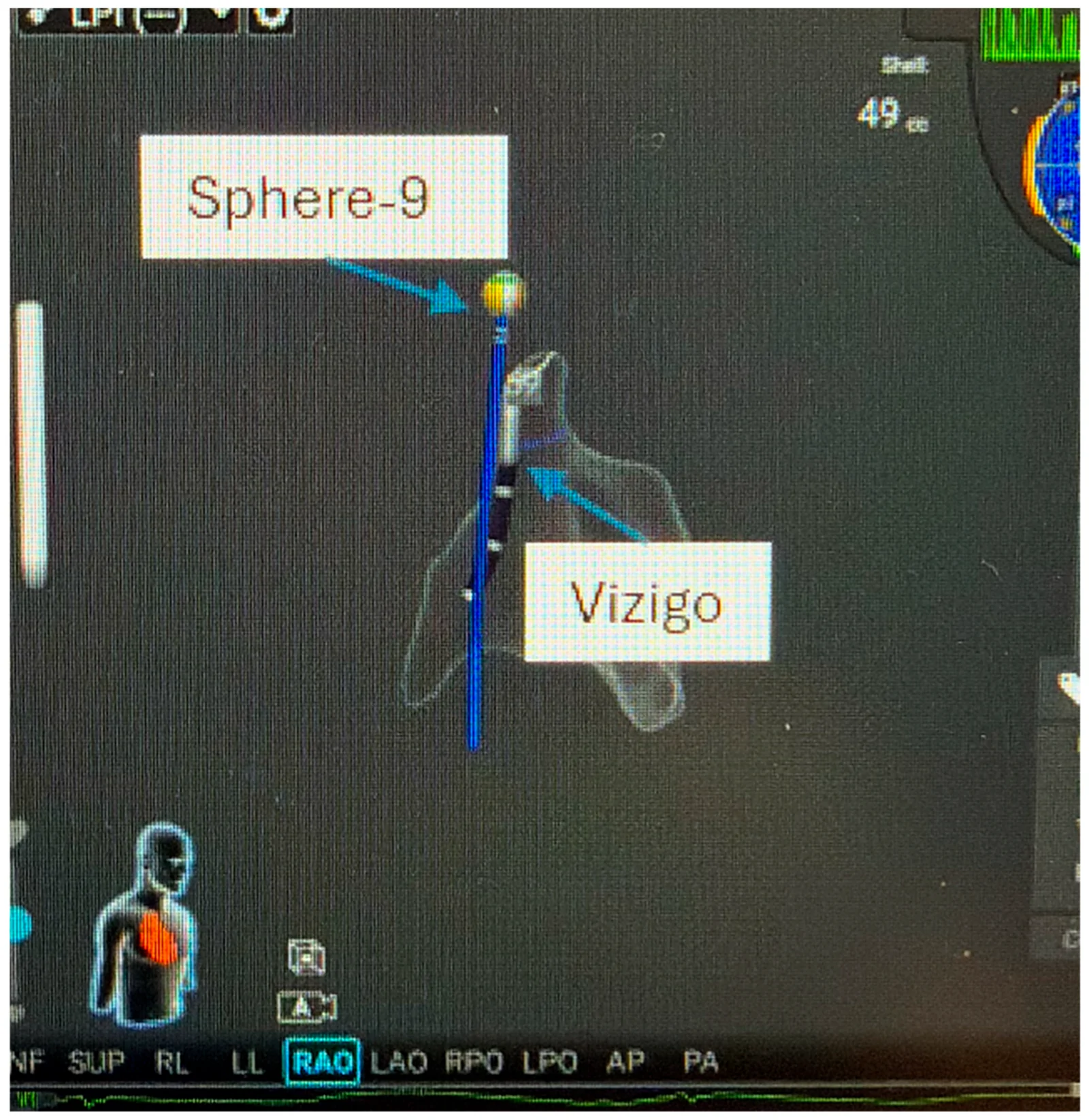
Simultaneous display of the Sphere-9 lattice-tip catheter and the VIZIGO bidirectional steerable sheath on the Affera three-dimensional map, obtained during a fluoroless procedure at the authors’ institution. The Affera/Prism system localizes devices by hybrid magnetic and calibrated-impedance (“synergy”) navigation and, per the manufacturer’s system documentation, provides visualization of non-sensor-based diagnostic catheters [75]. That documentation addresses diagnostic catheters rather than sheaths; no comparative outcome data on sheath visualization exist for this platform, and the image is presented as an illustration of our workflow.

**Step 6: Right atrial mapping [D/I].** Before transseptal puncture, the Sphere-9 catheter is advanced into the RA through the VIZIGO sheath, and a three-dimensional reconstruction of the RA is created on the Affera mapping system (Figure 8). The SVC, SVC-RA junction, IVC-RA junction, His bundle region, tricuspid annulus, CS ostium, and the low-voltage region corresponding to the fossa ovalis are identified and tagged (Figure 9). The CS catheter is advanced into the coronary sinus and displayed on the map as a stable anatomical and pacing reference. The Sphere-9 catheter is then advanced into the SVC and the VIZIGO sheath advanced over it, the sheath being localized by the converging methods described in Step 4; in our procedures it can be appreciated on the Affera map in relation to the Sphere-9 catheter (Figure 10). Transseptal puncture is performed as described in Steps 4 and 5.

**Figure 11:**
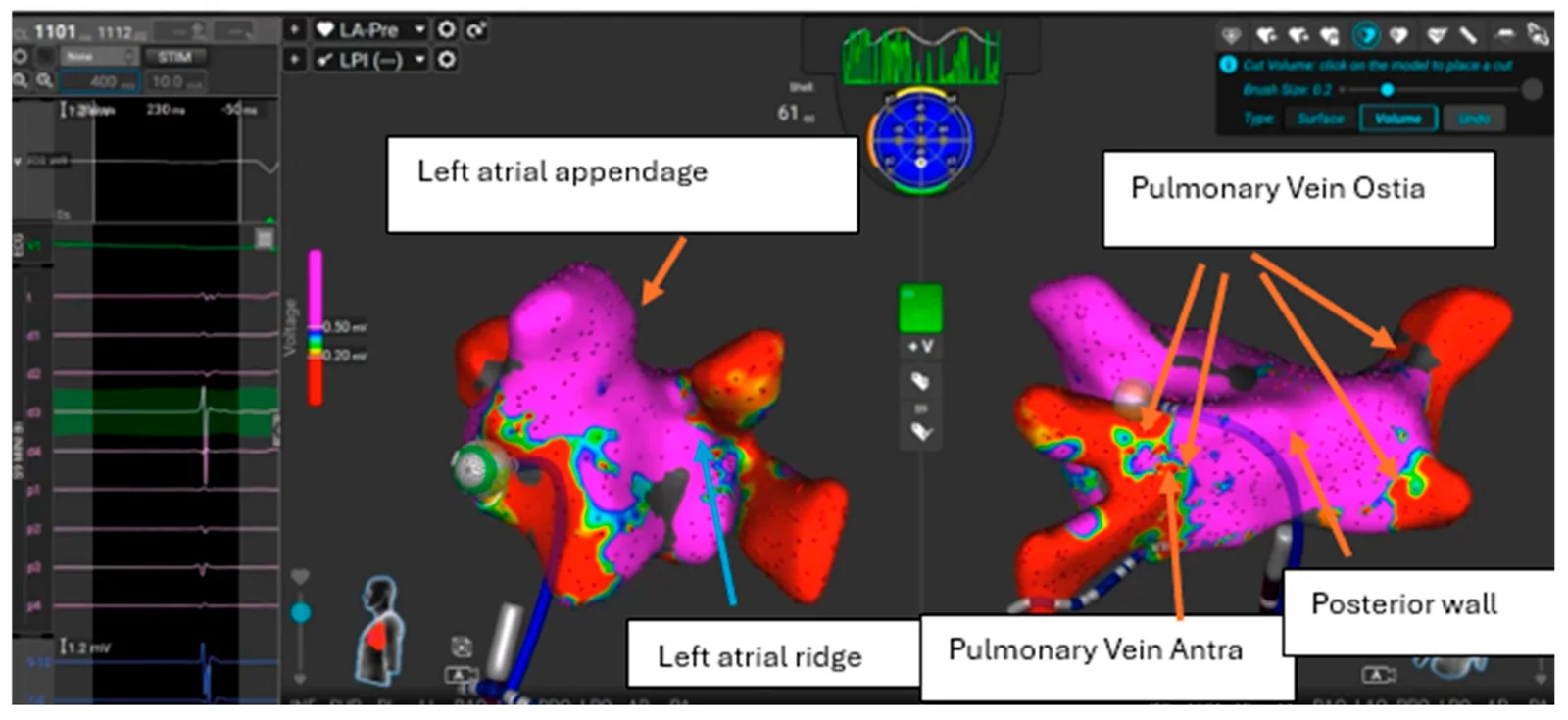
High-density left atrial and pulmonary vein voltage map acquired with the Sphere-9 lattice-tip catheter on the Affera mapping system. The nine mini-electrodes and integrated sensors enable delineation of the: left atrial appendage, pulmonary vein ostia, left atrial ridge, pulmonary vein antra, and posterior wall, with real-time impedance-based contact assessment.

**Step 7: Left atrial mapping [D].** Following transseptal access, the Sphere-9 catheter is advanced into the LA. Using the nine mini-electrodes, embedded thermocouples, and integrated magnetic sensor, a high-density voltage and anatomical map of the LA and PVs is created. The ostia and antra of all four PVs, the LAA, the left atrial ridge, and the posterior wall are delineated. Impedance-based feedback is used to assess tissue contact and optimize catheter position during both mapping and lesion delivery (Figure 11).

**Figure 12:**
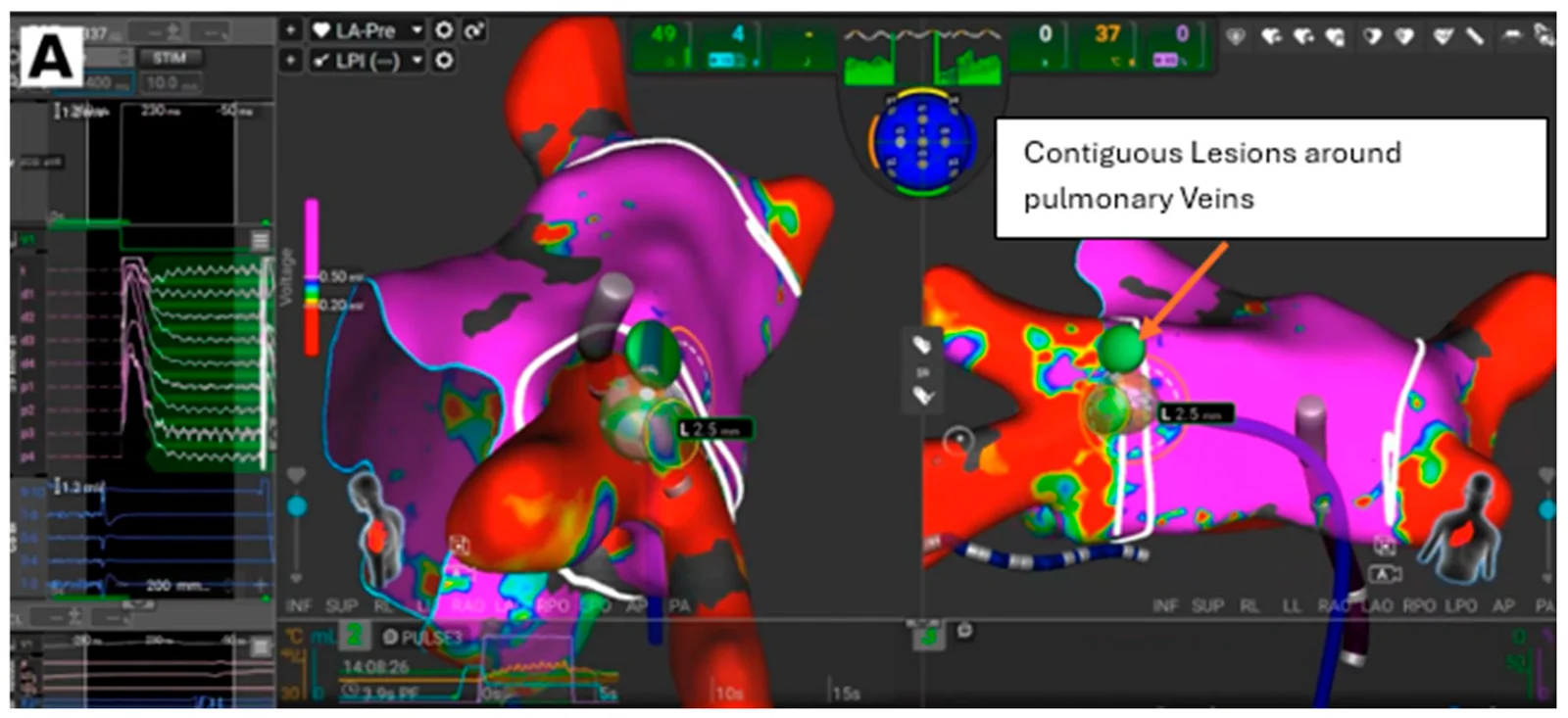

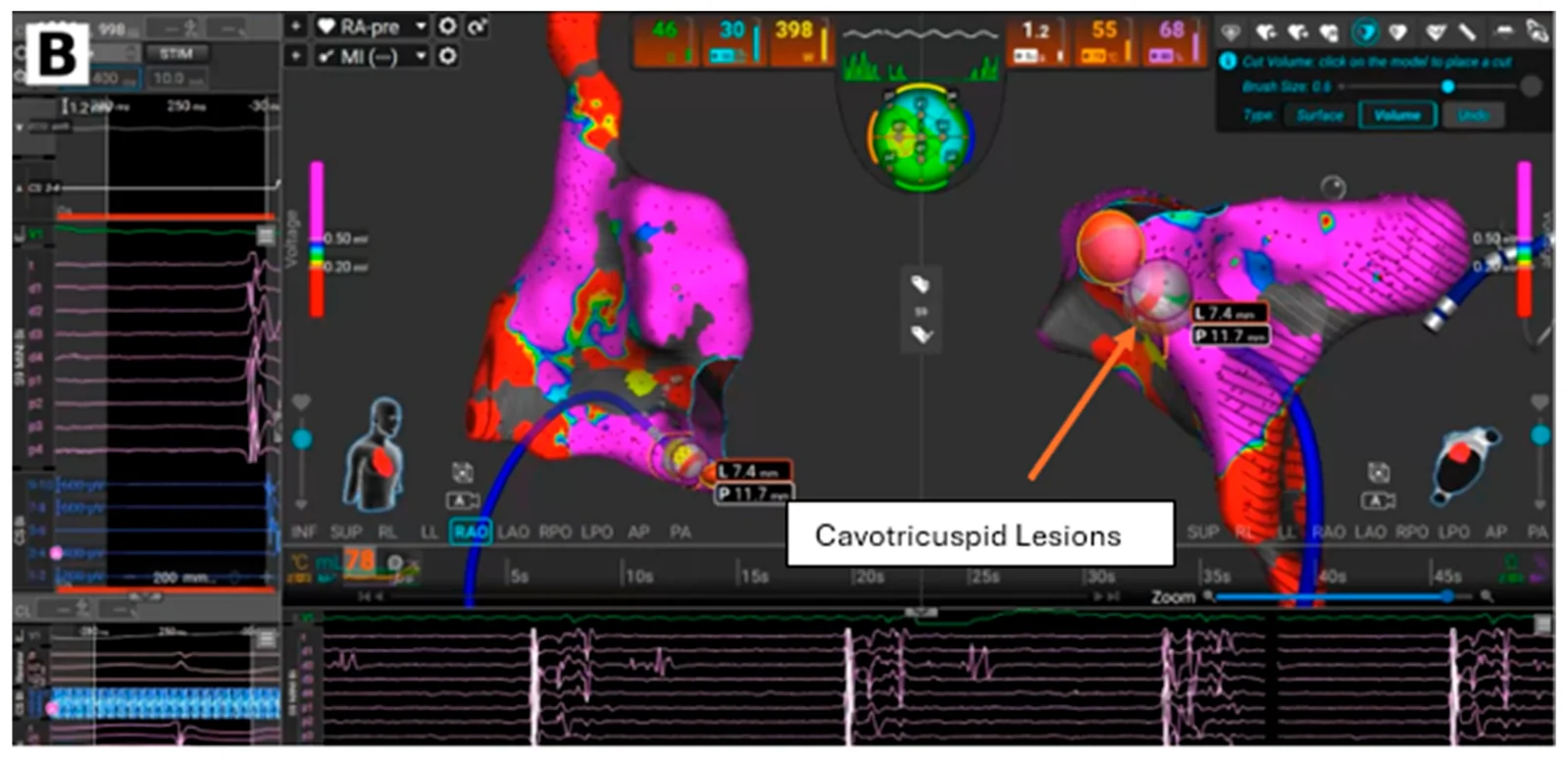
Dual-energy lesion delivery with the Sphere-9 lattice-tip catheter. (**A**) Pulsed field applications delivered circumferentially around the pulmonary vein antra, with real-time impedance-based contact indication. (**B**) Completed cavotricuspid isthmus line created with the same catheter without catheter exchange, using both energy sources: temperature-controlled radiofrequency applications (red tags) in the segment adjacent to the tricuspid annulus, where pulsed field energy is avoided, and pulsed field applications (green tags) along the remainder of the isthmus toward the inferior vena cava. The yellow ring marks the currently selected lesion tag. Images were obtained on the Affera mapping system during a procedure performed at the authors’ institution.

**Step 8: Pulmonary vein isolation and contact assessment [D].** PVI is performed using point-by-point biphasic pulsed field energy delivered through the lattice tip, with applications placed circumferentially around each PV antrum and contiguous lesion placement. Before energy delivery, catheter–tissue apposition is judged from two signals on the Affera system: impedance-based contact indication, with the local impedance rise displayed on the beat graph and on the electrode signals; and dynamic sphere highlighting on the three-dimensional map, in which individual electrodes illuminate as contact improves. The embedded thermocouples do not contribute to this pre-delivery assessment. During temperature-controlled radiofrequency applications the thermocouples provide closed-loop temperature control; during pulsed field applications the recorded temperature change is small and serves only as an adjunctive intraprocedural or post-application marker of energy–tissue interaction. Applications with inadequate pre-delivery contact indication, or with an unexpected temperature response on review after delivery, are repeated (Figure 12).

**Step 9: Confirmation of pulmonary vein isolation [D].** Entrance and exit block are assessed using the mapping electrodes of the Sphere-9 catheter, and the PV antra are remapped to identify residual conduction gaps, which are targeted with further applications until acute bidirectional block is confirmed. As above, acute block is not evidence of durable isolation in the absence of provocative testing or repeat mapping at a later date.

**Step 10: Additional lesion sets and energy selection [D/I].** The dual-energy capability permits seamless transition between pulsed field and temperature-controlled RF energy without catheter exchange. In our practice, pulsed field energy is used preferentially for PVI and posterior wall isolation, along the posterior LA wall and the inferior aspects of the inferior PVs, where radiofrequency is avoided. Temperature-controlled RF is used in the immediate vicinity of the mitral and tricuspid annuli, where pulsed field energy is avoided. Linear lesions—the mitral isthmus line, the roof line and the CTI line—are created with whichever energy source suits each segment. Cavotricuspid isthmus ablation is therefore performed as a hybrid line with the same catheter and without catheter exchange: temperature-controlled radiofrequency adjacent to the tricuspid annulus and pulsed field energy along the remainder of the isthmus toward the inferior vena cava (Figure 12B). Bidirectional isthmus block is confirmed by differential pacing. This approach matches the energy source to the anatomical substrate and intended lesion set and is consistent with the lesion strategy employed in the SPHERE Per-AF trial [7,59].

**Step 11: Post-ablation assessment and vascular closure [I].** ICE is performed to assess for pericardial effusion, intracardiac thrombus, and other complications, protamine is administered to reverse anticoagulation, and sheath removal and vascular closure are performed as described for the VARIPULSE workflow.

## 10. Comparison of the Two Systems

Table 1 summarizes the technical and evidentiary differences between the two platforms. The effectiveness values are drawn from different trials in different populations and are presented for orientation only; they must not be read as a head-to-head comparison.

Sphere-9 and VARIPULSE are not the only mapping-integrated pulsed field ablation catheters in clinical use, and the field is now multi-vendor. The FARAWAVE NAV catheter (Boston Scientific) is magnetically tracked and integrates with the OPAL HDx mapping system through the FARAVIEW software module, providing dynamic visualization of catheter placement, shape and rotation together with real-time contact information and supporting fluoroscopy reduction; an average fluoroscopy time of 3.8 min was reported in the DISRUPT-AF registry (n = 477) [76]. Navigation-enabled pulsed field platforms from other manufacturers are likewise in clinical use or under evaluation. This review is confined to the Sphere-9 and VARIPULSE systems because these are the two mapping-integrated pulsed field platforms with which the authors have direct procedural experience and because a step-by-step fluoroless workflow is only worth describing where the authors can vouch for each step. The general principles set out here—right atrial electroanatomical mapping before transseptal access, tagging of anatomical landmarks, intracardiac echocardiographic guidance of transseptal puncture, catheter-first sheath advancement, and explicit criteria for reverting to fluoroscopy—are not platform-specific and should transfer to any mapping-integrated pulsed field system, though we have not tested them on other platforms and do not present them as validated there.

## 11. Zero-Fluoroscopy PFA: Current Evidence

Evidence specific to fluoroless PFA is accumulating rapidly but remains observational. A systematic review of fluoroscopy-free PFA synthesized five studies comprising 260 patients and reported 100% acute PVI in all studies, a fluoroscopy time of 0 min in four of five studies, freedom from AF of 72.1–80%, and no cardiac tamponade in any fluoroscopy-free cohort [58]. A larger meta-analysis by Erzinger et al. pooled 14 observational studies comprising 1005 patients and found a pooled acute success rate of 100% (95% CI 1.0–1.0; I2 = 0%), a low prevalence of complications, a mean procedure time of 80.9 min (95% CI 64.9–97.1), and a mean left atrial dwell time of 46 min (95% CI 32.0–61.5) [77]. Single-center series have reported fully fluoroless PFA workflows with comparable results [78].

The most informative dataset on procedural efficiency comes from a high-volume academic center that analyzed 827 consecutive zero-fluoroscopy first-time ablations performed between January 2022 and March 2025. Zero-fluoroscopy RF and PFA procedures were 25 and 14 min shorter, respectively, than fluoroscopy-guided procedures (*p* < 0.001). Within the zero-fluoroscopy cohort, PFA was 25 min faster than RF overall, with the advantage most pronounced for PVI plus posterior wall isolation (−28 min) compared with PVI alone (−16 min). Critically for adoption, the learning curve for zero-fluoroscopy PFA did not vary by physician or by years of experience and plateaued after 114 total PFA cases [74]. That the learning curve is independent of career stage is the single most practically encouraging finding for centers contemplating a fluoroless transition.

Taken together with the AdmIRE fluoroscopy subgroup analysis [65] and the randomized sheath-visualization trial [32], these findings indicate that fluoroless PFA is feasible, efficient, and safe in experienced hands, while falling short of the evidence standard that would justify recommending it universally. A recent narrative review reaches similar conclusions across the broader range of zero-fluoroscopy procedures [79]. Published dedicated fluoroscopy-free and near-fluoroscopy-free PFA studies are summarised in Table 2.

## 12. Limitations and Future Directions

Several limitations of the present evidence base, and of this review, should be stated plainly. First, the zero-fluoroscopy PFA literature is dominated by observational, single-center studies with modest sample sizes, and no randomized trial has compared zero-fluoroscopy PFA with fluoroscopy-guided PFA. The randomized evidence that exists addresses adjacent questions—sheath visualization [32] or PFA versus thermal energy or drug therapy [5,7,46,47]—rather than the radiation strategy itself. The best available fluoroscopy-stratified data are a non-randomized, physician-discretion subgroup analysis [65]. Second, long-term follow-up for zero-fluoroscopy PFA is limited, and durability data beyond 12 months are sparse. Third, ICE adds cost and is not universally available, particularly in parts of Europe; workflows dependent on it are not universally transferable, although the SHORT LOOK registry demonstrates that an EAM-only route can achieve near-zero fluoroscopy [41]. Formal cost-effectiveness analyses of fluoroless strategies are lacking. Fourth, the crossover rate from zero-fluoroscopy to conventional fluoroscopy, while low at 1.26% [36], is not zero, and feasibility in the most rigorous AF-specific analysis was 95.1% rather than 100% [38]. A fluoroless program requires fluoroscopy to remain immediately available. Fifth, this is a narrative review. Study selection was structured but not systematic; no risk-of-bias assessment or quantitative pooling was performed, and selection bias in the choice of included studies cannot be excluded. Several of the pivotal studies cited are industry-sponsored, including the trials of both catheters that are the subject of this review, and the AdmIRE fluoroscopy analysis received manufacturer medical-writing support. Sixth, the workflow described in Section 9 reflects a single institution’s practice with particular equipment and has not been prospectively validated. Its transferability to centers with different sheaths, ICE catheters, or mapping platforms is untested.

Seventh, an asymmetry in the evidence deserves emphasis. Dedicated fluoroless outcome data exist for the VARIPULSE platform, including a fluoroscopy-stratified analysis with 12-month outcomes and several prospective or consecutive series [62,65,66,70]. No equivalent series exists for the Sphere-9 platform, for which reported fluoroscopy exposure is low (approximately 4.4 ± 3.1 min) rather than zero [62]. As set out in Section 7.3, we regard the fluoroless Sphere-9 workflow as a reasoned extension of a shared and evidenced enabling stack rather than a validated protocol, but the distinction is material and readers should weigh the two workflows accordingly. A prospective consecutive series of fluoroless Sphere-9 ablation reporting fluoroscopy time, acute pulmonary vein isolation, complications, and same-day discharge would resolve this directly and is the single most useful study the field could add on this question.

Eighth, the comparative literature on which this review draws does not include pre-specified non-inferiority designs or margins with respect to fluoroscopy strategy, and the absence of a detected difference should not be equated with demonstrated non-inferiority. Ninth, much of the dose-comparison literature predates contemporary ultra-low-dose fluoroscopy systems, so the incremental benefit of a fluoroless strategy over a modern low-dose protocol is smaller than historical comparisons suggest.

Tenth, the scope of this review is narrower than the field it describes. Mapping-integrated pulsed field ablation is multi-vendor, and platforms other than the two considered here—including the FARAWAVE NAV catheter with the OPAL HDx mapping system and FARAVIEW module [76]—support comparable navigation-based workflows (Section 10). We have confined this review to the Sphere-9 and VARIPULSE systems because these are the platforms with which we have direct procedural experience, and the workflow described should not be read as a comparative endorsement of these two systems over others.

Promising future directions include nanosecond PFA potentially enabling conscious sedation, artificial-intelligence-assisted ICE reconstruction to shorten mapping time [29,72], four-dimensional volumetric ICE, extension of fluoroless PFA to non-PV and ventricular targets, standardized definitions of “zero fluoroscopy” to permit meaningful pooling, dedicated fluoroless evaluation of the Sphere-9 platform, and adequately powered randomized comparison of fluoroless versus conventional PFA workflows.

## 13. Conclusions

Pooled comparative studies report similar acute success, arrhythmia recurrence and complication rates between zero-fluoroscopy and fluoroscopy-guided AF ablation, and PFA has been compared with thermal energy, cryoballoon ablation and antiarrhythmic drug therapy in randomized trials and in a registry of more than 40,000 patients. Neither body of evidence was designed to validate a fluoroscopy-free procedural strategy, and the randomized data show no consistent reduction in fluoroscopy time attributable to PFA itself.

What the evidence does support is narrower and, we would argue, more useful. The reduction in radiation is achieved by the enabling stack rather than the energy source: electroanatomical mapping, intracardiac echocardiography, ICE-guided transseptal puncture, and above all direct visualization of the steerable sheath, for which randomized evidence shows that left atrial fluoroscopy time falls to zero. The native mapping integration of the Sphere-9 and VARIPULSE catheters makes them well suited to this stack. A non-randomized subgroup analysis of AdmIRE found similar 12-month freedom from AF across zero-, low- and conventional-fluoroscopy strategies, and the learning curve for zero-fluoroscopy PFA appears independent of career stage, which lowers the barrier to adoption.

The workflow described here should be understood as an expert- and experience-based approach, supported by limited and largely observational evidence, and not as an established standard of care. It requires appropriate patient selection, adequate operator and laboratory experience, and explicit bailout criteria, with fluoroscopy immediately available and used without hesitation when indicated. Randomized comparison of fluoroless versus conventional PFA, dedicated evaluation of the Sphere-9 platform, and a standardized definition of “zero fluoroscopy” are the necessary next steps. Until then, patient safety rather than the absolute avoidance of fluoroscopy must remain the governing principle.

## Figures and Tables

**Table 1:** Comparison of the Sphere-9 and VARIPULSE platforms.

Feature	Sphere-9 (Affera/Medtronic)	VARIPULSE (Biosense Webster)
Catheter design	9 mm lattice-tip, focal, dual-energy	Variable-loop circular (25–35 mm adjustable)
Energy modality	Dual (PF + temperature-controlled RF)	PF only
Mapping system	Affera (Medtronic)	CARTO 3
Ablation approach	Point-by- point	Circumferential
Contact/proximity sensing	Local impedance + thermocouple temperature feedback	Tissue proximity indication (TPI; unipolar impedance)
Pivotal trial	SPHERE Per-AF (persistent AF, randomized vs. RF)	AdmIRE (paroxysmal AF; single-arm pivotal)
12- month effectiveness	73.8%	74.6%
Zero-fluoroscopy rate	17.7% (<0.5 min), 45.8% ≤5 min	>25% (first-quartile 0.0 min)
Dedicated fluoroless outcome data	None published	Yes- multiple consecutive series, fluoroscopy-stratified AsmIRE analysis
Principal design characteristic	Dual energy enables RF/PF toggle without catheter exchange	Native CARTO 3/TPI integration; single catheter maps and ablates

AF, atrial fibrillation; CF, conventional fluoroscopy; LF, low fluoroscopy; PF, pulsed field; RF, radiofrequency; ZF, zero fluoroscopy. IMPORTANT: the effectiveness values in this table are not comparable. SPHERE Per-AF was a randomized trial in persistent AF with an active comparator, whereas AdmIRE was a single-arm pivotal study in paroxysmal AF; the two report different endpoints in different populations, and the numerical similarity of the two percentages is coincidental. No head-to-head randomized comparison of these catheters exists. This table contrasts device design, workflow characteristics, and the nature of the available evidence, not clinical superiority.

**Table 2:** Published studies of dedicated fluoroscopy-free or near-fluoroscopy-free PFA for atrial fibrillation.

Study, Year	Design and Population	PFA Catheter/Mapping System	AF Phenotype	Imaging Guidance	Definition of Near/Zero Fluoroscopy; Fluoroscopy Time	Crossover to Fluoroscopy	Acute Outcome and Procedure Time	Safety and Follow-Up
Chan et al., Pacing Clin Electrophysiol 2026 [66]	Prospective single-center consecutive series (n = 34)	VARIPULSE/CARTO 3	Paroxysmal and persistent AF	ICE + CARTO 3; no pre-procedural CT/MRI; 2 sheaths only	Zero fluoroscopy—no fluoroscopy used in any case	0/34	100% acute PVI; median procedure time 40.5 min	No major complications; peri-procedural follow-up
Teumer et al., J Clin Med 2025 [67]	Prospective single-center workflow study (n = 10; 3 conventional, 7 “mapping-on-the-fly”)	VARIPULSE/CARTO 3 v8 with VIZIGO sheath	Paroxysmal and persistent AF	TEE (no ICE) + CARTO 3	Zero fluoroscopy—all procedures completed without fluoroscopy	0/10	100% acute PVI; median procedure time 144 min (conventional) vs. 68 min (optimized), *p* = 0.017	No acute complications attributable to the zero-fluoroscopy technique; one ischemic stroke ~10 h post-procedure, mild outcome
Yamashita et al., Pacing Clin Electrophysiol 2025 [71]	Prospective single-center workflow study	PulseSelect circular multielectrode PFA catheter (Medtronic)/3D EAM—not VARIPULSE	Paroxysmal AF	ICE + 3D EAM	Near-zero fluoroscopy—0.1 min, limited to initial registration	Not reported	100% acute PVI; mean total procedure time 48.5 min (left PV 9.0 min, right PV 11.0 min)	No significant procedural complications; low recurrence on 3-month Holter
Sawalha et al., Pacing Clin Electrophysiol 2026 [58]	Systematic review (5 studies, 260 patients)	Mixed PFA platforms	Mixed	Mixed—ICE and/or 3D EAM	Fluoroscopy-free; fluoroscopy time 0 min in 4 of 5 studies	Not reported	100% acute PVI in all included studies	No cardiac tamponade in any fluoroscopy-free cohort; freedom from AF 72.1–80%
Erzinger et al., J Interv Card Electrophysiol 2026 [77]	Systematic review and meta-analysis (14 observational studies, 1005 patients)	Mixed PFA platforms	Mixed	Mixed	Zero or low fluoroscopy; definitions varied between the included studies	Not reported	Pooled acute success 100% (95% CI 1.0–1.0; I^2^ = 0%); mean procedure duration 80.9 min (95% CI 64.9–97.1); mean LA dwell time 46 min (95% CI 32.0–61.5)	Low pooled prevalence of complications; low arrhythmia recurrence on descriptive analysis
Pérez-Pinzón et al., Heart Rhythm 2025 [74]	Retrospective single-center consecutive cohort (827 zero-fluoroscopy ablations)	Mixed—PFA compared with RF	Mixed	ICE + 3D EAM	Zero fluoroscopy	Not reported	Zero-fluoroscopy PFA 25 min shorter than zero-fluoroscopy RF ablation (*p* < 0.001); learning curve plateau after ~114 PFA cases, independent of career stage	Severe complications 0.48%, equally rare in both groups

AF, atrial fibrillation; CI, confidence interval; EAM, electroanatomical mapping; ICE, intracardiac echocardiography; LA, left atrial; PFA, pulsed field ablation; PV, pulmonary vein; PVI, pulmonary vein isolation; RF, radiofrequency; TEE, transesophageal echocardiography. When a study did not report an item, the cell reads “Not reported” rather than being left blank. The SHORT LOOK registry [41] is not included in this table: it is a near-zero-fluoroscopy atrial fibrillation ablation registry performed deliberately without intracardiac echocardiography and without a non-fluoroscopic tracking system and is not a pulsed field ablation cohort; it is cited in Section 5.2 among the general zero-fluoroscopy evidence [41]. The studies differ in design, platform, phenotype and definition of zero fluoroscopy and must not be read as a head-to-head comparison.

## Data Availability

No new data were created or analyzed in this study. Data sharing is not applicable to this article.
